# Genome-Wide Association Study Using Extreme Truncate Selection Identifies Novel Genes Affecting Bone Mineral Density and Fracture Risk

**DOI:** 10.1371/journal.pgen.1001372

**Published:** 2011-04-21

**Authors:** Emma L. Duncan, Patrick Danoy, John P. Kemp, Paul J. Leo, Eugene McCloskey, Geoffrey C. Nicholson, Richard Eastell, Richard L. Prince, John A. Eisman, Graeme Jones, Philip N. Sambrook, Ian R. Reid, Elaine M. Dennison, John Wark, J. Brent Richards, Andre G. Uitterlinden, Tim D. Spector, Chris Esapa, Roger D. Cox, Steve D. M. Brown, Rajesh V. Thakker, Kathryn A. Addison, Linda A. Bradbury, Jacqueline R. Center, Cyrus Cooper, Catherine Cremin, Karol Estrada, Dieter Felsenberg, Claus-C. Glüer, Johanna Hadler, Margaret J. Henry, Albert Hofman, Mark A. Kotowicz, Joanna Makovey, Sing C. Nguyen, Tuan V. Nguyen, Julie A. Pasco, Karena Pryce, David M. Reid, Fernando Rivadeneira, Christian Roux, Kari Stefansson, Unnur Styrkarsdottir, Gudmar Thorleifsson, Rumbidzai Tichawangana, David M. Evans, Matthew A. Brown

**Affiliations:** 1 University of Queensland Diamantina Institute, University of Queensland, Princess Alexandra Hospital, Brisbane, Australia; 2 Medical Research Council Centre for Causal Analyses in Translational Epidemiology, University of Bristol, Bristol, United Kingdom; 3 Academic Unit of Bone Metabolism, Metabolic Bone Centre, University of Sheffield, Sheffield, United Kingdom; 4 The University of Melbourne, Department of Clinical and Biomedical Sciences: Barwon Health, Geelong, Australia; 5 School of Medicine and Pharmacology, University of Western Australia, Perth, Australia; 6 Department of Endocrinology and Diabetes, Sir Charles Gairdner Hospital, Perth, Australia; 7 Garvan Institute of Medical Research, Sydney, Australia; 8 St. Vincent's Clinical School, St. Vincent's Hospital Campus, University of New South Wales, Sydney, Australia; 9 Menzies Research Institute, University of Tasmania, Hobart, Australia; 10 Kolling Institute, Royal North Shore Hospital, University of Sydney, Sydney, Australia; 11 Department of Medicine, University of Auckland, Auckland, New Zealand; 12 Medical Research Council Lifecourse Epidemiology Unit, Southampton, United Kingdom; 13 University of Melbourne Department of Medicine and Bone and Mineral Service, Royal Melbourne Hospital, Melbourne, Australia; 14 Departments of Medicine, Human Genetics, Epidemiology and Biostatistics, Lady Davis Institute, Jewish General Hospital, McGill University, Montreal, Canada; 15 Department of Twin Research and Genetic Epidemiology, King's College London, London, United Kingdom; 16 Department of Internal Medicine and Epidemiology, Erasmus Medical Center, Rotterdam, The Netherlands; 17 Medical Research Council Mammalian Genetics Unit, Harwell Science and Innovation Campus, Harwell, Oxfordshire, United Kingdom; 18 Academic Endocrine Unit, Nuffield Department of Clinical Medicine, Oxford Centre for Diabetes, Endocrinology, and Metabolism, University of Oxford, Churchill Hospital, Headington, Oxford, United Kingdom; 19 National Institute for Health and Research Biomedical Research Unit, University of Oxford, Oxford, United Kingdom; 20 Centre of Muscle and Bone Research, Charité – University Medicine Berlin, Campus Benjamin Franklin, Free and Humboldt University, Berlin, Germany; 21 Medizinische Physik, Klinik für Diagnostische Radiologie, Universitätsklinikum Schleswig-Holstein, Kiel, Germany; 22 School of Medicine, Deakin University, Geelong, Australia; 23 Department of Endocrinology and Diabetes, Barwon Health, Geelong, Australia; 24 Institute of Bone Joint Research, University of Sydney, Royal North Shore Hospital, Sydney, Australia; 25 School of Public Health and Community Medicine, University of New South Wales, Sydney, Australia; 26 Division of Applied Medicine, University of Aberdeen, Aberdeen, United Kingdom; 27 Rheumatology Department, AP-HP Cochin Hospital – Paris-Descartes University, Paris, France; 28 deCODE Genetics, Reykjavik, Iceland; 29 University of Iceland, Reykjavik, Iceland; Georgia Institute of Technology, United States of America; David B. Allison, University of Alabama at Birmingham, United States of America

## Abstract

Osteoporotic fracture is a major cause of morbidity and mortality worldwide. Low bone mineral density (BMD) is a major predisposing factor to fracture and is known to be highly heritable. Site-, gender-, and age-specific genetic effects on BMD are thought to be significant, but have largely not been considered in the design of genome-wide association studies (GWAS) of BMD to date. We report here a GWAS using a novel study design focusing on women of a specific age (postmenopausal women, age 55–85 years), with either extreme high or low hip BMD (age- and gender-adjusted BMD z-scores of +1.5 to +4.0, n = 1055, or −4.0 to −1.5, n = 900), with replication in cohorts of women drawn from the general population (n = 20,898). The study replicates 21 of 26 known BMD–associated genes. Additionally, we report suggestive association of a further six new genetic associations in or around the genes *CLCN7, GALNT3, IBSP, LTBP3, RSPO3*, and *SOX4*, with replication in two independent datasets. A novel mouse model with a loss-of-function mutation in *GALNT3* is also reported, which has high bone mass, supporting the involvement of this gene in BMD determination. In addition to identifying further genes associated with BMD, this study confirms the efficiency of extreme-truncate selection designs for quantitative trait association studies.

## Author Summary

Osteoporotic fracture is a major cause of early mortality and morbidity in the community. To identify genes associated with osteoporosis, we have performed a genome-wide association study. In order to improve study power and to address the demographic group of highest risk from osteoporotic fracture, we have used a unique study design, studying 1,955 postmenopausal women with either extreme high or low hip bone mineral density. We then confirmed our findings in 20,898 women from the general population. Our study replicated 21 of 26 known osteoporosis genes, and it identified a further six novel loci (in or nearby *CLCN7, GALNT3, IBSP, LTBP3, RSPO3*, and *SOX4*). For one of these loci, *GALTN3*, we demonstrate in a mouse model that a loss-of-function genetic mutation in *GALNT3* causes high bone mass. These findings report novel mechanisms by which osteoporosis can arise, and they significantly add to our understanding of the aetiology of the disease.

## Introduction

Osteoporotic fracture is a leading cause of morbidity and mortality in the community, particularly amongst the elderly. In 2004 ten million Americans were estimated to have osteoporosis, resulting in 1.5 million fractures per annum [1]. Hip fracture is associated with a one year mortality rate of 36% in men and 21% in women [2]; and the burden of disease of osteoporotic fractures overall is similar to that of colorectal cancer and greater than that of hypertension and breast cancer [3]. Bone mineral density (BMD) is strongly correlated with bone strength and fracture risk, and its measurement is widely used as a diagnostic tool in the assessment of fracture risk [4]–[6]. BMD is known to be highly heritable, with heritability assessed in both young and elderly twins, and in families, to be 60–90% [7]–[14]. Although the extent of covariance between BMD and fracture risk is uncertain, of the 26 genes associated with BMD at genome-wide significant levels to date, nine have been associated with fracture risk (reviewed in [15]), supporting the use of BMD as an intermediate phenotype in the search for genes associated with fracture risk.

There is considerable evidence from genetic studies in humans [12], [16], [17], and in mice [18], indicating that the genes that influence BMD at different sites, and in the different genders, overlap but are not identical. Thus far all genome-wide association studies (GWAS) of BMD have studied cohorts of a wide age range, and with one exception have included both men and women; when only women have been studied, both pre- and postmenopausal women have been included. Therefore, to identify genes involved in osteoporosis in the demographic at highest risk of osteoporotic fracture we have performed a GWAS in postmenopausal women selected on the basis of their hip BMD, and replicated the GWAS findings in a large cohort of adult women drawn from the general population.

## Results

Considering markers previously reported as associated with BMD, our discovery dataset replicates previously associated SNPs in 21 of the 26 genes reported to date to have genome-wide significant associations (Table S6) (P<0.05, association in the same direction as initially reported, or, in the case of *LRP5* and *GPR177,* with the next flanking SNP genotyped) [17], [21], [22], [23], [28], [32], [33], [34]. Replicated genes include *ARHGAP1, CTNNB1, ESR1, FAM3C, FLJ42280, FOXL1, GPR177, HDAC5, JAG1, LRP5, MARK3, MEF2C, MEPE, OPG, RANK, RANKL, SOST, SOX6, SP7* (Osterix), *STARD3NL* and *ZBTB40*. Considering the combined Anglo-Australasian Osteoporosis Genetics Consortium (AOGC) and deCODE/TwinsUK/Rotterdam cohorts, 97 SNPs from six loci achieved P<5×10^−8^ at the femoral neck (FN), of which four had previously been reported (*FLJ42280, MEF2C, SOX6, ZBTB40*). At the lumbar spine (LS), six SNPs from two known loci (*RANKL, OPG)* achieved P<5×10^−8^. No support was seen for previously reported associations involving SNPs in *ADAMTS18, CRHR1, DCDC5, MHC,* or *SBTBN1* (P>0.05).

This study also identifies and replicates two novel loci with confirmed association with BMD in *GALNT3* (MIM: 601756) and at chromosome 6q22 near *RSPO3* (MIM: 610574), and provides strong evidence of a further four BMD-associated loci (*CLCN7* (MIM: 602727), *IBSP* (MIM: 147563), *LTBP3* (MIM: 602090), *SOX4* (MIM: 184430)) (Table 1). Although these did not achieve ‘genome-wide significance’ in the discovery set alone, they achieved P-values in the AOGC-discovery cohort of P<10^−4^, and support in the AOGC-replication cohort, TwinsUK, Rotterdam and deCODE cohorts; and all have additional evidence supporting their role in bone. Support was also seen for *TGFBR3* (MIM: 600742), a gene previously reported to have suggestive association with BMD [33].

**Table 1:** Findings for novel replicated associations.

				AOGC DISCOVERY						REPLICATION				COMBINED DISCOVERY/REPLICATION			
				TH		FN		LS		FN		LS		FN		LS	
LOCUS	SNP	A1/A2	GENE	BETA	P-VALUE	BETA	P-VALUE	BETA	P-VALUE	BETA	P-VALUE	BETA	P-VALUE	BETA	P-VALUE	BETA	P-VALUE
2q24	rs1863196	A/G	*GALNT3*	0.284	2.3×10^-5^	−0.090	3.7×10^−5^	−0.068	0.037	−0.065	0.0011	−0.01	0.72	−0.077	2.0×10^−7^	−0.024	0.16
2q24	rs6710518	C/T	*GALNT3*	0.262	6.9×10^−5^	−0.078	0.0015	−0.068	0.039	−0.057	1.2×10^−6^	−0.037	0.01	−0.064	4.8×10^−10^	−0.042	0.0017
4q22	rs1054627	A/G	*IBSP*	0.277	6.6×10^−5^	−0.042	0.00024	−0.049	0.18	−0.043	9.2×10^−5^	−0.027	0.046	−0.050	7.6×10^−7^	−0.03	0.019
6p22	rs9466056	A/G	*SOX4*	−0.237	5.3×10^−4^	0.090	6.6×10^−5^	0.10	0.0036	0.033	0.0033	0.021	0.17	0.049	4.2×10^−6^	0.035	0.014
6q22	rs17563605	T/C	*RSPO3*	0.30	2.1×10^−4^	−0.10	7.4×10^−5^	−0.088	0.020	−0.051	2.1×10^−4^	−0.047	0.0097	−0.062	2.5×10^−7^	−0.055	0.00082
6q22	rs13204965	A/C	*RSPO3*	0.30	2.1×10^−4^	−0.10	7.3×10^−5^	−0.089	0.020	−0.057	3.5×10^−5^	−0.049	0.0082	−0.067	3.0×10^−8^	−0.056	0.00067
11p13	rs1152620	A/G	*LTBP3*	−0.311	4.4×10^−5^	0.060	0.020	0.080	0.041	0.039	0.0051	0.013	0.48	0.044	3.6×10^−4^	0.025	0.13
16p13	rs13336428	A/G	*CLCN7*	−0.221	7.0×10^−4^	0.057	0.013	0.076	0.028	0.040	0.0011	0.045	0.0050	0.044	5.1×10^−5^	0.051	5.1×10^−4^

Findings for novel replicated associations for the AOGC discovery and replication sets, combined replication sets (AOGC replication/TwinsUK/Rotterdam/deCODE) and entire dataset (AOGC discovery and replication/TwinsUK/Rotterdam/deCODE). The regression coefficient in the TH analysis shows the expected increase in the log odds ratio of low BMD per addition of allele A2. The regression coefficients in the FN and LS analyses refer to the expected increase in standardized BMD per addition of allele A2.

### *GALNT3*

**Figure 1:**
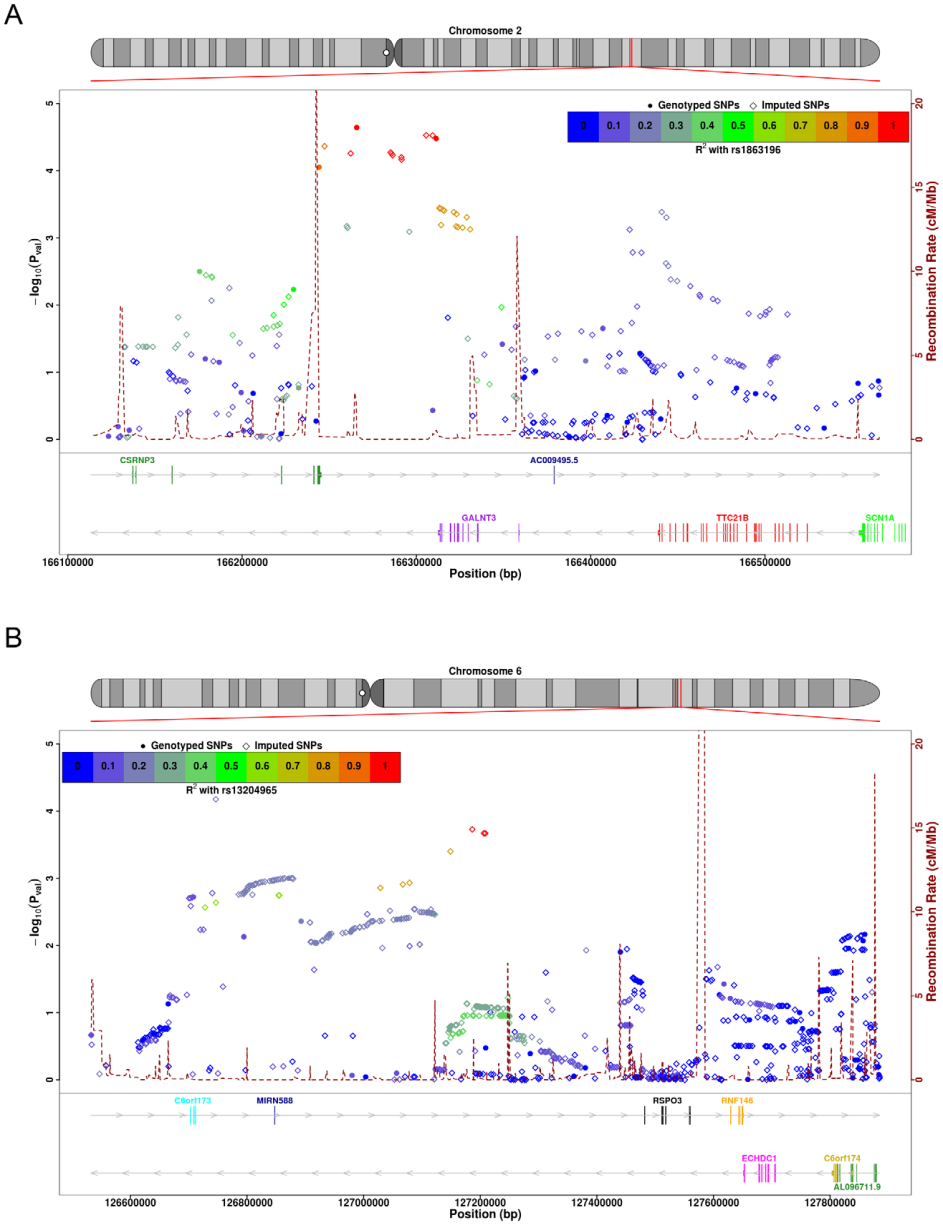
SNP association plots for BMD-associated regions. Discovery cohort association significance level is plotted against the left hand y-axis as -log10(P-values). Genetic coordinates are as per NCBI build 36.1. Filled circles represent genotyped SNPs, and outlined diamonds represent imputed SNPs. The recombination rate (cM/Mb as per HapMap data) is indicated by the purple dotted line and right hand y-axis. Genes and ESTs are indicated with their approximate sizes and direction of translation. (A) Chromosome 2q24 - *GALNT3* region. SNP association plot of findings from TH case-control analysis of AOGC discovery set for a 500 kb region (166,100 kb to 166,600 kb) of chromosome 2. LD is indicated by colour scale in relationship to marker rs1863196. (B) Chromosome 6q22 - *RSPO3* region. SNP association plot of findings from TH case-control analysis of AOGC discovery set for a 1,200 kb region (126,600 kb to 127,800 kb) of chromosome 6. LD is indicated by colour scale in relationship to marker rs13204965.

SNPs at chromosome 2q24, in and around *GALNT3*, achieved near genome-wide significance in our discovery cohort (peak P-value rs1863196, total hip (TH) P = 2.3×10^−5^; LS P = 0.037) (Figure 1A). This SNP was not typed or imputed by either the Rotterdam or the TwinsUK cohorts, but a nearby SNP showed strong association in both AOGC and the combined replication cohorts (rs6710518; AOGC discovery, TH P = 6.9×10^−5^; combined replication sets, FN P = 2.7×10^−7^). In the combined datasets the finding achieved genome-wide significance at the FN (P = 1.7×10^−10^). Strong association was also seen with this SNP at LS (P = 7.5×10^−5^). Another marker within *GALNT3*, rs4667492, was also associated with fracture risk, including vertebral fractures (OR = 0.89; 95%CI = 0.80–0.99; P = 0.032) and overall low trauma fractures (OR = 0.92; 95%CI = 0.85–0.99; P = 0.024).

**Figure 2:**
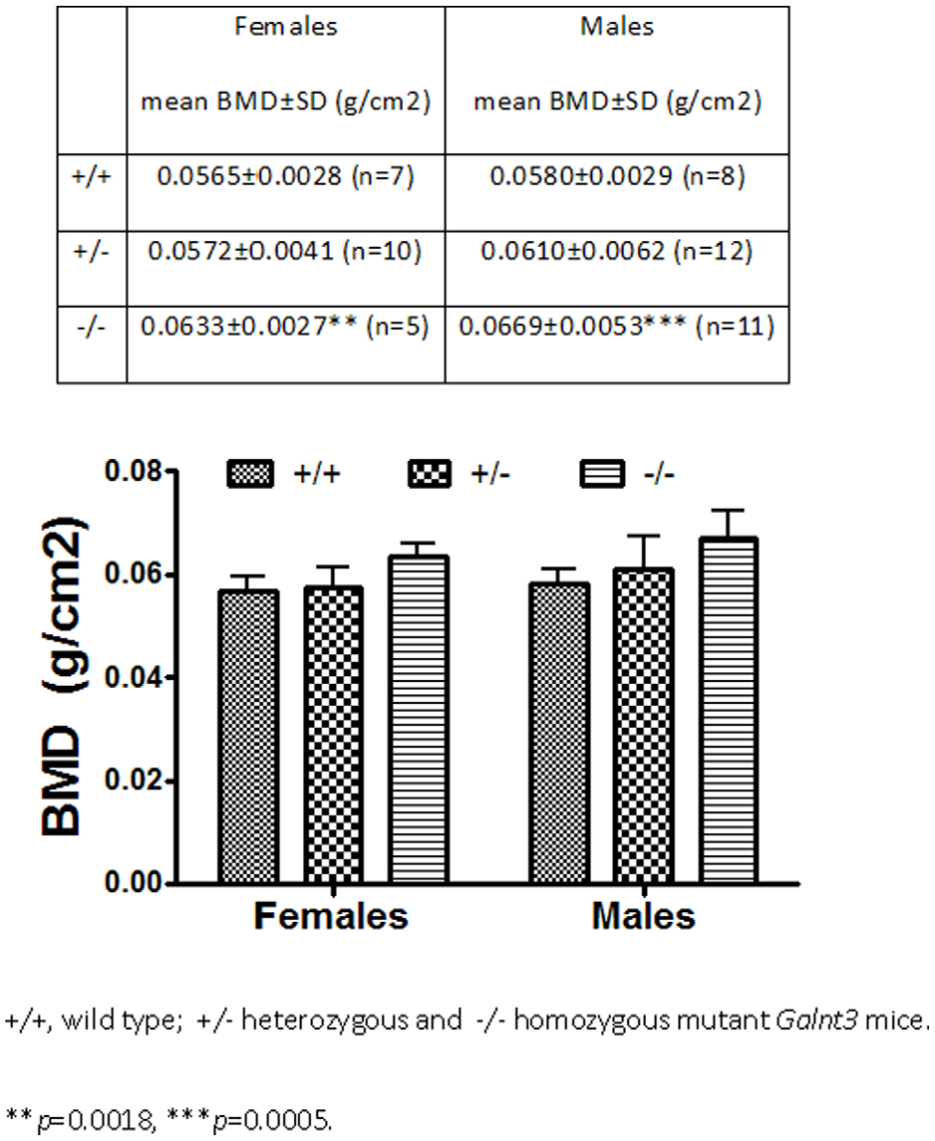
Areal BMD derived from DEXA analysis of 15- to 16-week-old *GALNT3* mutant and wild-type mice. P-values refer to Student t-test for two-way ANOVA across the three genotypes.

We have recently identified a mouse with an *N*-ethyl-*N*-nitrosourea induced loss-of-function *GALNT3* mutation (Trp589Arg), that develops hyperphosphataemia with extraskeletal calcium deposition, and hence represents a model for FTC [35]. To establish further the association of *GALNT3* and BMD, we determined BMD in these *GALNT3* mutant mice. This revealed that homozygous (−/−) *GALNT3* mutant male and female adult mice had a higher areal BMD than their wild-type (+/+) litter mates, with heterozygous (+/−) mice having intermediate BMD (Figure 2). This loss-of-function *GALNT3* mutation is predicted to lead to a reduced glycosylation of FGF23, which increases its breakdown and leads to reduced serum FGF23 concentrations [35].

### *RSPO3*

A novel genome-wide significant association was also seen at markers on chromosome 6q22-23 (Figure 1B). In the combined dataset, marker rs13204965 achieved genome-wide significance at this locus at the FN (P = 2.2×10^−9^), with strong support in both the AOGC discovery set, and the combined replication sets (AOGC-discovery, TH P = 2.1×10^−4^; combined replication P = 3.5×10^−5^). Strong association was also seen with LS BMD (rs13204965 P = 0.00067). The peak of association at this locus lies within a cDNA fragment, AK127472. The nearest gene, *RSPO3* (R-spondin-3), is 275 kb telomeric of the strongest associated SNP, but is within the associated linkage disequilibrium region (Figure 1B).

### *CLCN7*

Association was observed at chromosome 16p13 with SNPs in and around *CLCN7*, which encodes a Cl^−^/H^+^ antiporter expressed primarily in osteoclasts, and critical to lysosomal acidification, an essential process in bone resorption. Peak association at this locus was seen with SNP rs13336428 in the discovery set (TH P = 7.0×10^−4^; LS P = 0.028) (Figure S3A), which was confirmed in the replication set (FN P = 3.6×10^−5^; LS P = 0.00012), achieving P = 1.7×10^−6^ at the FN and 1.2×10^−5^ at LS in the overall cohort. Association has previously been reported between two SNPs in exon 15 of *CLCN7* (rs12926089, rs12926669) and FN BMD (P = 0.001–0.003) [36]; no association was seen with either of those SNPs in the current study (P>0.4 at FN and LS).

### *IBSP*

Association was observed with SNPs in *IBSP* (integrin-binding bone sialoprotein) (Figure S3B), encoded at chromosome 4q22, a gene which has previously had suggestive association reported with BMD in two studies (rs1054627, Styrkarrsdottir et al P = 4.6×10^−5^ [22]; Koller et al P = 1.5×10^−4^ [37]). In the current study, moderate association was observed in the discovery set with the same SNP as previously reported (rs1054627, AOGC discovery TH, P = 6.6×10^−5^), with support in the replication set and strong association overall (FN combined replication P = 9.2×10^−5^; FN overall association P = 7.6×10^−7^). Nominal association was observed at LS (rs1054627, P = 0.019).

### *LTBP3*

Association with BMD was also seen at chromosome 11p13, with SNP rs1152620 achieving P = 4.4×10^−5^ (TH) in the discovery set, P = 0.0051 (FN) in the replication set, and P = 3.6×10^−4^ overall (Figure S3C). This SNP was also nominally associated with LS BMD in the discovery set (P = 0.041). The nearest gene to this locus is *LTBP3* (latent transforming growth factor beta binding protein 3), which is located 292 kb q-telomeric of rs1152620.

### *SOX4*

At chromosome 6p22, SNPs in and around *SOX4* (Sex determining region Y box 4) were moderately associated with BMD in our discovery set (most significant association rs9466056, TH P = 5.3×10^−4^; LS P = 0.0036) (Figure S3D), with support at the hip and LS in the replication set (FN P = 0.00013, LS P = 0.013), achieving association overall with P = 2.6×10^−7^ (FN) and P = 0.00081 (LS).

## Discussion

This study demonstrates convincing evidence of association with six genes with BMD variation, *GALNT3, RSPO3, CLCN7, IBSP, LTBP3* and *SOX4*. Using a moderate sample size, the use of a novel study design also led to the confirmation of 21 of 26 known BMD-associations. This study thus demonstrates the power of extreme-truncate selection designs for association studies of quantitative traits.

*GALNT3* encodes N-acetylgalactosaminyltransferase 3, an enzyme involved in 0-glycosylation of serine and threonine residues. Mutations of *GALNT3* are known to cause familial tumoral calcinosis (FTC, OMIM 2111900) [38] and hyperostosis-hyperphosphataemia syndrome (HOHP, OMIM 610233) [39]. FTC is characterised by hyperphosphataemia in association with the deposition of calcium phosphate crystals in extraskeletal tissues; whereas in HOHP, hyperphosphataemia is associated with recurrent painful long bone swelling and radiographic evidence of periosteal reaction and cortical hyperostosis. *FGF23* mutations associated with FTC cause hyperphosphataemia through effects on expression of the sodium-phosphate co-transporter in the kidney and small intestine, and through increased activation of vitamin D due to increased renal expression of CYP27B1 (25-hydroxyvitamin-D 1 alpha hydroxylase) [40]. It is unclear whether FGF23 has direct effects on the skeleton or if its effects are mediated through its effects on serum phosphate and vitamin D levels. FGF23 signals via a complex of an FGF receptor (FGFR1(IIIc)) and Klotho [41]; mice with a loss-of-function mutation in *Klotho* develop osteoporosis amongst other abnormalities, and modest evidence of association of *Klotho* with BMD has been reported in several studies [42], [43], [44], [45]. We saw no association with polymorphisms in *Klotho* and BMD in the current study (P>0.05 for all SNPs in and surrounding *Klotho*). To our knowledge, this finding is the first demonstration in humans that genetic variants in the FGF23 pathway are associated with any common human disease.

*RSPO3* is one of four members of the R-spondin family (R-spondin-1 to −4), which are known to activate the Wnt pathway, particularly through effects on LRP6, itself previously reported to be BMD-associated [46], [47]. LRP6 is inhibited by the proteins Kremen and DKK1, which combine to induce endocytosis of LRP6, reducing its cell surface levels. R-spondin family members have been shown to disrupt DKK1-dependent association of LRP6 and Kremen, thereby releasing LRP6 from this inhibitory pathway [48]. R-spondin-4 mutations cause anonychia (absence or severe hypoplasia of all fingernails and toenails, OMIM 206800) [49]. No human disease has been associated with R-spondin-3, and knockout of R-spondin-3 in mice is embryonically lethal due to defective placental development [50].

Mutations of *CLCN7* cause a family of osteopetroses of differing age of presentation and severity, including infantile malignant *CLCN7*-related recessive osteopetrosis (ARO), intermediate autosomal osteopetrosis (IAO), and autosomal dominant osteopetrosis type II (ADOII, Albers-Schoenberg disease). These conditions are characterized by expanded, dense bones, with markedly reduced bone resorption. Our data support associations of polymorphisms at this locus with BMD variation in the population.

IBSP is a major non-collagenous bone matrix protein involved in calcium and hydroxyapatite binding, and is thought to play a role in cell-matrix interactions through RGD motifs in its amino acid sequence. IBSP is expressed in all major bone cells including osteoblasts, osteocytes and osteoclasts; and its expression is upregulated in osteoporotic bone [51]. *IBSP* knockout mice have low cortical but high trabecular bone volume, with impaired bone formation, resorption, and mineralization [52]. *IBSP* lies within a cluster of genes including *DMP1, MEPE*, and *SPP1,* all of which have known roles in bone and are strong candidate genes for association with BMD. *MEPE* has previously been associated with BMD at genome-wide significance [17]. In the current study the strongest association was seen with an SNP in *IBSP,* rs1054627, as was the case with two previous studies [22], [37]. Linkage disequilibrium between this SNP, and the previously reported BMD-associated SNP rs1471403 in *MEPE*, is modest (r^2^ = 0.16). Whilst out study supports the association of common variants in *IBSP* in particular with BMD, further studies will be required to determine if more than one of these genes is BMD-associated.

Recessive mutations of *LTBP3* have been identified as the cause of dental agenesis in a consanguineous Pakistani family (OMIM 613097) [53]. Affected family members had base of skull thickening, and elevated axial but not hip BMD. *LTBP3−/−* mice develop axial osteosclerosis with increased trabecular bone thickness, as well as craniosynostosis [54]. LTBP3 is known to bind TGFβ1, -β2 and -β3, and may influence chondrocyte maturation and enchondral ossification by effects on their bioavailability [54].

Our study also confirms the previously reported association of another TGF pathway gene, *TGFBR3*, encoded at chromosome 1p22, with BMD [33] (Figure S3E). In that study, association was observed in four independent datasets, but overall the findings did not achieve genome-wide significance at any individual SNP (most significant SNP rs17131547, P = 1.5×10^−6^). In our discovery set, peak association was seen at this locus with SNP rs7550034 (TH P = 1.5×10^−4^), which lies 154 kb q-telomeric of rs17131547, but still within *TGFBR3* (rs17131547 was not typed or imputed in our dataset) (Figure S3E). This supports *TGFBR3* as a true BMD-associated gene.

This study also demonstrated that *SOX4* polymorphisms are associated with BMD variation. Both *SOX4* and *SOX6* are cartilage-expressed transcription factors known to play essential roles in chondrocyte differentiation and cartilage formation, and hence endochondral bone formation. *SOX6* has previously been reported to be BMD-associated at genome-wide significant levels [17]. Whilst *SOX4*−/− mice develop severe cardiac abnormalities and are non-viable, *SOX4+/−* mice have osteopaenia with reduced bone formation but normal resorption rates, and diminished cortical and trabecular bone volume [55]. Our data suggest that *SOX4* polymorphisms contribute to the variation in BMD in humans.

This study has a unique design amongst GWAS of BMD reported to date, using an extreme-truncate ascertainment scheme, focusing on a specific skeletal site (TH), and with recruitment of a narrow age- and gender-group (post-menopausal women age 55–85 years). Our goal in employing this scheme was to increase the study power by reducing heterogeneity due to age-, gender- and skeletal site-specific effects. Whilst osteoporotic fracture can occur at a wide range of skeletal sites, hip fracture in postmenopausal women is the major cause of morbidity and mortality due to osteoporosis. To date, with only one exception, all GWAS of BMD have studied cohorts unselected for BMD [28], and no study has restricted its participants to postmenopausal women ascertained purely on the basis of hip BMD. Assuming marker-disease-associated allele linkage disequilibrium of r^2^ = 0.9, for alpha = 5×10^−8^ our study has 80% power to detect variants contributing 0.3% of the additive genetic variance of BMD. An equivalent-powered cohort study would require ∼16,000 unselected cases.

Considering the 26 known genes (or genomic areas) associated with BMD, P-values less than <0.05 were seen in our discovery for 21 of the BMD-associated SNPs. Of the 26 known BMD genes, 16 would have been included in our replication study on the basis of the strength of their BMD association in our discovery cohort, but were not further genotyped as they were known already to be BMD-associated. Had these 16 genes replicated, 22 genes would have been identified in this single study, demonstrating the power of the design of the current study.

A potential criticism of studies of highly selected cohorts, such as the AOGC-discovery cohort, is that the associations identified may not be relevant in the general population. However, the confirmation of our findings in replication cohorts of women unselected for BMD confirms that our findings are of broad relevance.

In summary, our study design therefore represents a highly efficient model for future studies of quantitative traits and is one of the first reported studies using an extreme truncate design in any disease. We have identified two new BMD loci at genome-wide significance (*GALNT3, RSPO3*), with *GALNT3* SNPs also associated with fracture. Strong evidence was also demonstrated for four novel loci (*CLCN7, IBSP*, *LTBP3, SOX4*). Further support was also provided that *TGFBR3* is a true BMD-associated locus. Our discovery cohort replicated 21 of 26 previously identified BMD-associated loci. Our novel findings further advance our understanding of the aetiopathogenesis of osteoporosis, and highlight new genes and pathways not previously considered important in BMD variation and fracture risk in the general population. Our study also provides strong support that the use of extreme truncate selection is an efficient and powerful approach for the study of quantitative traits.

## Materials and Methods

### Ethics statement

All participants gave written, informed consent, and the study was approved by the relevant research ethics authorities at each participating centre.

### Subjects and phenotypes

The discovery sample population included 1128 Australian, 74 New Zealand and 753 British women, between 55–85 years of age, five or more years postmenopausal, with either high BMD (age- and gender-adjusted BMD z-scores of +1.5 to +4.0, n = 1055) or low BMD (age- and gender-adjusted BMD z-scores of −4.0 to −1.5, n = 900) (Tables S1 and S2). BMD z-scores were determined according to the Geelong Osteoporosis Study normative range [19]. Low BMD cases were excluded if they had secondary causes of osteoporosis, including corticosteroid usage at doses equivalent to prednisolone ≥7.5 mg/day for ≥6 months, past or current anticonvulsant usage, previous strontium usage, premature menopause (<45 years), alcohol excess (>28 units/week), chronic renal or liver disease, Cushing's syndrome, hyperparathyroidism, thyrotoxicosis, anorexia nervosa, malabsorption, coeliac disease, rheumatoid arthritis, ankylosing spondylitis, inflammatory bowel disease, osteomalacia, and neoplasia (cancer, other than skin cancer). Screening blood tests (including creatinine (adjusted for weight), alkaline phosphatase, gamma-glutamyl transferase, 25-hydroxyvitamin D and PTH) were checked in 776 cases, and no differences were found between the high and low BMD groups. Therefore no further screening tests were done of the remaining cases.

Fracture data were analysed comparing individuals who had never reported a fracture after the age of 50 years, with individuals who had had a low or non-high trauma (low trauma fracture  =  fracture from a fall from standing height or less) osteoporotic fracture (excluding skull, nose, digits, hand, foot, ankle, patella) after the age of 50 years. Vertebral, hip and non-vertebral fractures were considered both independently and combined.

All participants were of self-reported white European ancestry.

DNA was obtained from peripheral venous blood from all cases except those recruited from New Zealand, for whom DNA was obtained from salivary samples using Oragene kits (DNA Genotek, Ontario, Canada). We have previously demonstrated that DNA from these two sources have equivalent genotyping characteristics [20].

After quality control checks including assessment of cryptic relatedness, ethnicity and genotyping quality, 900 individuals with low TH BMD and 1055 individuals with high TH BMD were available for analysis.

The replication cohort consisted of 8928 samples drawn from nine cohort studies, outlined in Tables S3 and S4 (‘AOGC replication cohort’) which were directly genotyped, These replication cases were adult women (age 20–95 years), unselected with regard to BMD, and who were not screened for secondary causes of osteoporosis. Replication was also performed in silico in 11,970 adult women from the TwinsUK and Rotterdam, and deCODE Genetics GWASs [21], [22], [23], in which association data were available at LS and FN.

High and low BMD ascertainment was defined according to the TH score, because this has better measurement precision than FN BMD [24]. However, neither TwinsUK nor the Rotterdam Study had TH BMD on the majority of their datasets and therefore were analysed using the FN measurement for which data were available on the whole cohort. All replication findings at the hip are reported therefore for FN BMD. TH and FN BMD are closely correlated (r = 0.882 in the AOGC dataset), with FN BMD one of the components of the TH BMD measurement.

### Genotyping

Genotyping of the discovery cohort (n = 2036) was performed using Illumina Infinium II HumHap300 (n = 140), 370CNVDuo (n = 4), 370CNVQuad (n = 1882) and 610Quad (n = 10) chips at the University of Queensland Diamantina Institute, Brisbane, Australia. Genotype clustering was performed using Illumina's BeadStudio software; all SNPs with quality scores <0.15 and all individuals with <98% genotyping success were excluded. 289499 SNPs were shared across all chip types. Cluster plots from the 500 most strongly associated loci, were manually inspected and poorly clustering SNPs excluded from analysis. Following imputation using the HapMap Phase 2 data, 2,543,887 SNPs were tested for association with TH and LS BMD (Manhattan plot of association findings, Figure S1). After data cleaning, minimal evidence of inflation of test statistics was observed, with a genomic inflation factor (λ) of 1.0282 (qq plot, Figure S2).

A total of 124 SNPs were successfully genotyped in the AOGC replication cohort. These replication study SNPs were selected from the findings of the discovery cohort, either based on the strength of association (P-value) or following analysis with GRAIL (n = 45) [25], using as seed data all SNPs previously reported to be associated with BMD at GWAS significant levels (results for all replication SNPs presented in Table S5). GRAIL is a bioinformatic program that assesses the strength of relationships between genes in regions surrounding input SNPs (usually derived from genetic association studies) and other SNPs or genes associated with the trait of interest, by assessing their co-occurrence in PubMed abstracts. Where genes surrounding input SNPs occur more frequently in abstracts with known associated genes, these SNPs are more likely themselves also to be associated, and can thus be prioritized for inclusion in replication studies.

For the replication study, genotyping was performed either by Applied Biosystems OpenArray (n = 113) or Taqman technology (n = 11) (Applied Biosystems, Foster City, CA, USA), according to the manufacturer's protocol.

### Statistical methods

Eleven individuals were removed because of abnormal X-chromosome homozygosity (X-chromosome homozygosity either <−0.14, or >+0.14). Outliers with regard to autosomal heterozygosity (either <0.34225 or >0.357, n = 40) and missingness (>3%, n = 4) were removed. Using an IBS/IBD analysis in PLINK to detect cryptic relatedness, one individual from 35 pairs of individuals with pi-hat >0.12 (equivalent to being 3^rd^ degree relatives or closer) were removed. SNPs with minor allele frequency <1% (n = 561), and those not in Hardy-Weinberg equilibrium (P<10^−7^, n = 170) were then removed, leaving 288,768 SNPs in total. Nine replication SNPs were removed because of excess missingness (>10%) or because they failed tests of Hardy-Weinberg equilibrium (P<0.001).

To detect and correct for population stratification EIGENSTRAT software was used. We first excluded the 24 regions of long range LD including the MHC identified in Price et al. before running the principal components analysis, as suggested by the authors [26]. Sixteen individuals were removed as ethnic outliers, leaving 1955 individuals in the final discovery dataset.

Imputation analyses were carried out using Markov Chain Haplotyping software (MaCH; http://www.sph.umich.edu/csg/abecasis/MACH/) using phased data from CEU individuals from release 22 of the HapMap project as the reference set of haplotypes. We only analyzed SNPs surrounding disease-associated SNPs that were either genotyped or could be imputed with relatively high confidence (R^2^≥0.3). For TH measurements, a case-control association analysis of imputed SNPs was performed assuming an underlying additive model and including four EIGENSTRAT eigenvectors as covariates, using the software package MACH2DAT [27] which accounts for uncertainty in prediction of the imputed data by weighting genotypes by their posterior probabilities. For FN and LS BMD analyses, Z-transformed residual BMD scores (in g/cm^2^) were generated for the entire AOGC cohort after adjusting for the covariates age, age^2^, and weight, and for centre of BMD measurement. Because the regression coefficient for BMD on genotype would be biased by selection for extremes, we adopted the approach detailed in Kung et al (2009) [28]. Specifically, the regression coefficient of genotype on BMD was estimated, and subsequently transformed to the regression coefficient of BMD on genotype through knowledge of the population variance of the phenotype and the allele frequencies. For fracture data, analysis was by logistic regression. Only SNPs achieving GWAS significance were tested for fracture association. The SNPs used for replication from the Rotterdam Study were analyzed using MACH2QTL implemented in GRIMP [29]. Data from the discovery and replication cohorts were combined using the inverse variance approach as implemented in the program METAL [30].

SNPs associated with BMD were also tested for association with fracture in the AOGC discovery and replication cohorts (hip, vertebral, nonvertebral, and all low trauma fractures, age ≥50 years, as defined above), by logistic regression.

Study power was calculated using the ‘Genetic Power Calculator’ [31].

### Mouse BMD analysis

All animal studies were approved by the MRC Harwell Unit Ethical Review Committee and are licensed under the Animal (Scientific Procedures) Act 1986, issued by the UK Government Home Office Department. Dual-energy X-ray absorptiometry (DEXA) was performed using a Lunar Piximus densitometer (GE Medical Systems) and analysed using the Piximus software.

### Data availability

Data related to this study will be available to research projects approved by a Data Access Committee including representatives of the University of Queensland Research Ethics Committee. For enquiries regarding access please contact the corresponding author, MAB (matt.brown@uq.edu.au).

## Supporting Information

Figure S1 Manhattan plot of discovery genome-wide association study findings for BMD at total hip. P = 10^−5^ is indicated by a blue horizontal line.
(0.51 MB TIF)

Figure S2 Genomic control findings. The genomic inflation factor (λ) when reported as the median χ^2^ was 1.0282.
(0.36 MB TIF)

Figure S3 SNP association plots for OP-associated regions. Discovery cohort association significance level is plotted against the left hand y-axis as -log10(P-values). Genetic coordinates are as per NCBI build 36.1. Filled circles represent genotyped SNPs, and outlined diamonds represent imputed SNPs. The recombination rate (cM/Mb as per HapMap data) is indicated by the purple dotted line and right hand y-axis. Genes and ESTs are indicated with their approximate sizes and direction of translation. (A) Chromosome 16p13 - *CLCN7* region. SNP association plot of findings from TH case-control analysis of AOGC discovery set for a 100 kb region (1,420 kb to 1,520 kb) of chromosome 16. LD is indicated by colour scale in relationship to marker rs13336428. (B) Chromosome 4q22 - *IBSP* region. SNP association plot of findings from TH case-control analysis of AOGC discovery set for a 500 kb region (88,700 kb to 89,200 kb) of chromosome 4. LD is indicated by colour scale in relationship to marker rs1054627. (C) Chromosome 11p13 - *LTBP3* region. SNP association plot of findings from TH case-control analysis of AOGC discovery set for a 300 kb region (64,950 kb to 65,250 kb) of chromosome 11. LD is indicated by colour scale in relationship to marker rs1152620. (D) Chromosome 6p22 - *SOX4* region. SNP association plot of findings from TH case-control analysis of AOGC discovery set for a 2 Mb region (20,500 kb to 22,500 kb) of chromosome 6. LD is indicated by colour scale in relationship to marker rs9466056. (E) Chromosome 1p22 - *TGFBR3* region. SNP association plot of findings from TH case-control analysis of AOGC discovery set for a 1 Mb region (91,800 kb to 92,800 kb) of chromosome 1. LD is indicated by colour scale in relationship to marker rs7550034.
(5.13 MB TIF)

Table S1 Case numbers for the discovery cohort, with BMD affection status and fracture history.
(0.05 MB DOC)

Table S2 Descriptive statistics for discovery cohort.
(0.06 MB DOC)

Table S3 Replication cohort details.
(0.04 MB DOC)

Table S4 Replication cohort fracture data.
(0.04 MB DOC)

Table S5 Replication study SNPs, beta coefficients and P-values for analysis of TH, FN and LS. The regression coefficient in the case-control analysis of TH in the discovery set shows the expected increase in the log odds ratio of low BMD per addition of allele A2. The regression coefficients in the TH, FN and LS analyses refer to the expected increase in standardized BMD per addition of allele A2 in the discovery set.
(0.22 MB DOC)

Table S6 Association findings in AOGC discovery set for markers achieving genome-wide significant association with BMD in previous studies. The regression coefficient in the TH analysis shows the expected increase in the log odds ratio of low BMD per addition of allele A2. The regression coefficients in the FN and LS analyses refer to the expected increase in standardized BMD per addition of allele A2.
(0.12 MB DOC)
