# Supplementary figures and images for: Genome-Wide Association Study Using Extreme Truncate Selection Identifies Novel Genes Affecting Bone Mineral Density and Fracture Risk
Source: PLoS Genet. 2011 Apr 21;7(4):e1001372. doi: 10.1371/journal.pgen.1001372 (PMC3080863; doi:10.1371/journal.pgen.1001372)

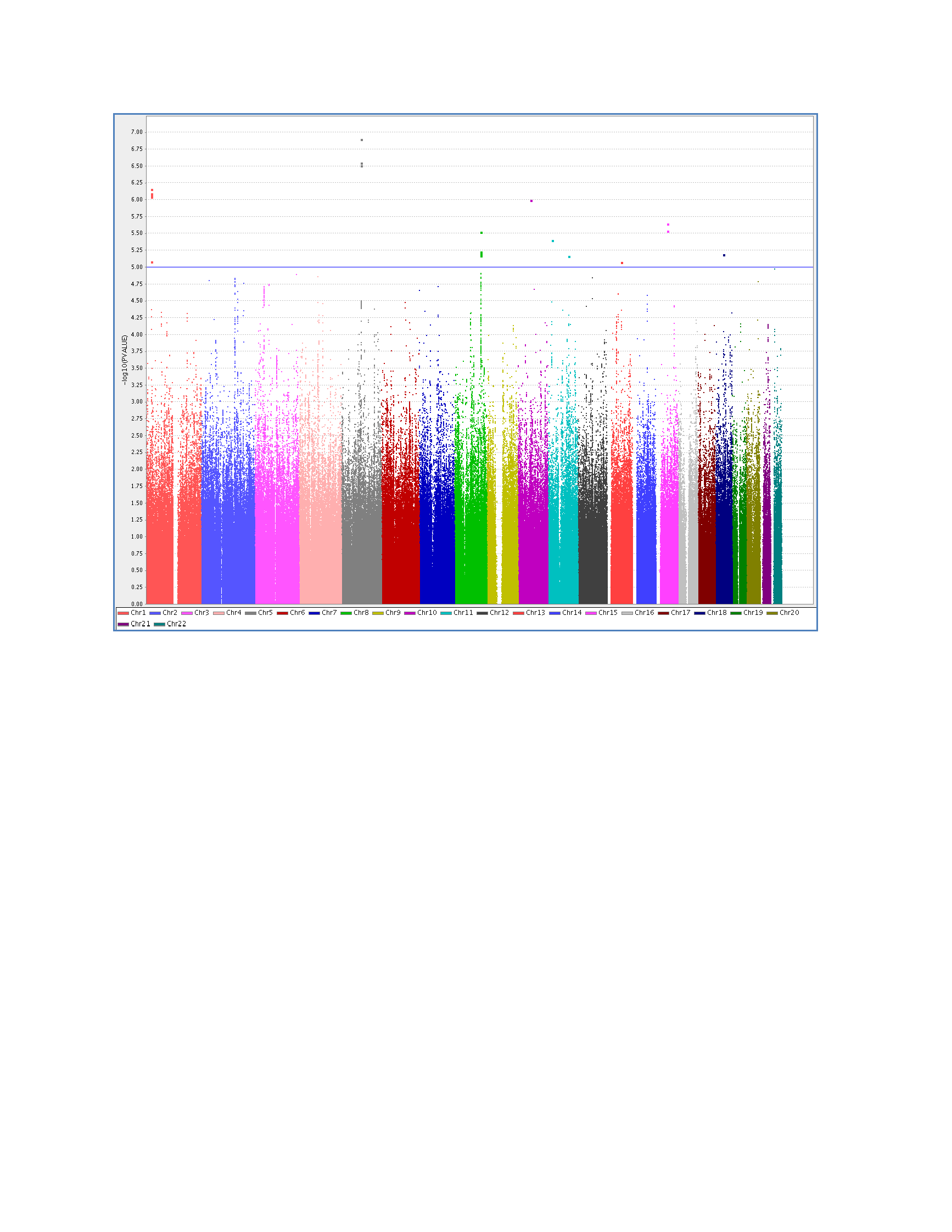

Supplement: Figure S1 — Manhattan plot of discovery genome-wide association study findings for BMD at total hip. P = 10−5 is indicated by a blue horizontal line. (0.51 MB TIF) [file pgen.1001372.s001.tif]

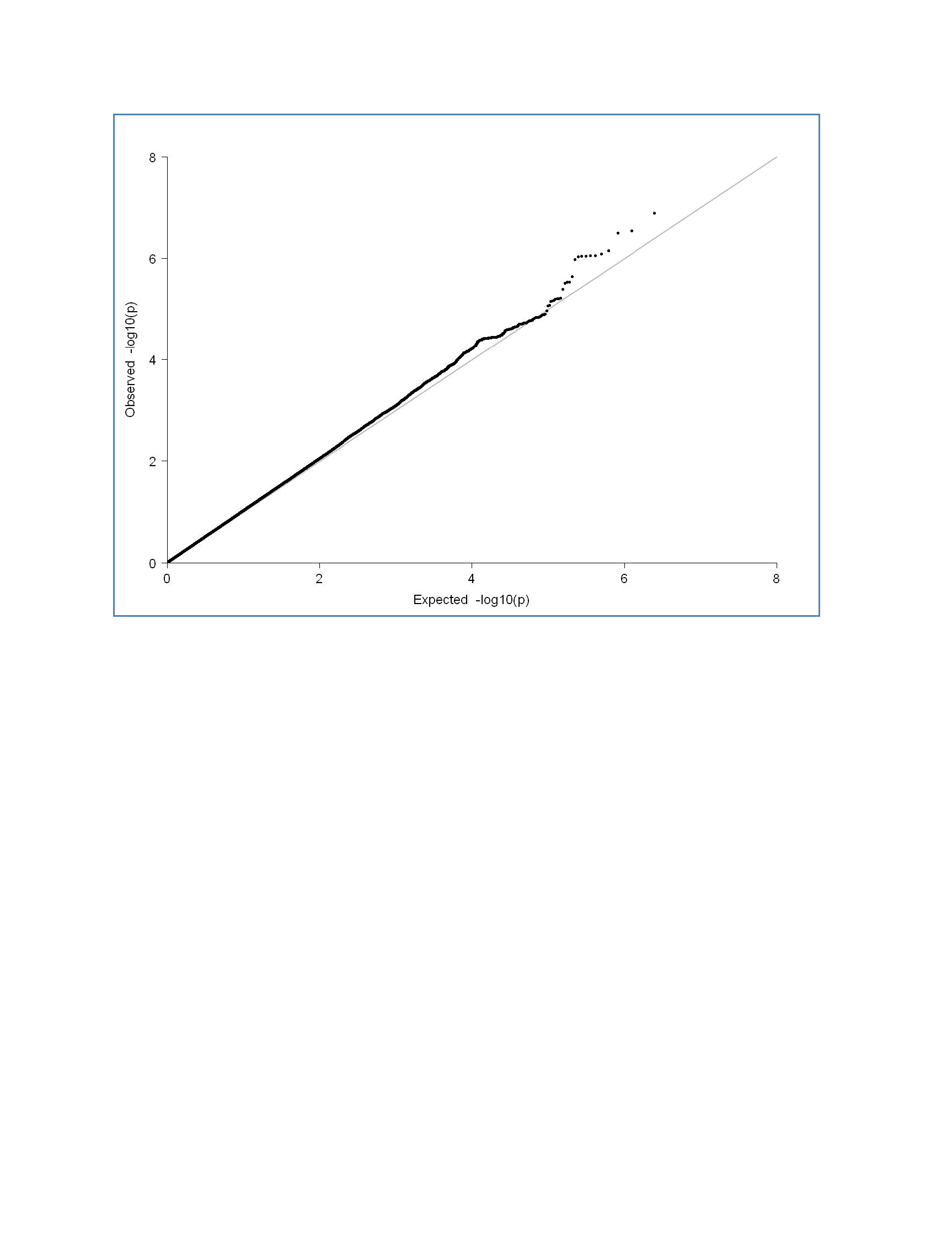

Supplement: Figure S2 — Genomic control findings. The genomic inflation factor (λ) when reported as the median χ2 was 1.0282. (0.36 MB TIF) [file pgen.1001372.s002.tif]
