# Supplementary material for: Genome-Wide Association Study Using Extreme Truncate Selection Identifies Novel Genes Affecting Bone Mineral Density and Fracture Risk
Source: PLoS Genet. 2011 Apr 21;7(4):e1001372. doi: 10.1371/journal.pgen.1001372 (PMC3080863; doi:10.1371/journal.pgen.1001372)
Supplement: Table S1 — Case numbers for the discovery cohort, with BMD affection status and fracture history. (0.05 MB DOC) [file pgen.1001372.s004.doc]

|  |  | CENTRE | | | | | | | | | | | |  |
| --- | --- | --- | --- | --- | --- | --- | --- | --- | --- | --- | --- | --- | --- | --- |
|  |  | Auckland  (NZ) | Brisbane  (Aust) | Dubbo  (Aust) | Geelong  (Aust) | Hertfordshire  (UK) | Hobart  (Aust) | Melbourne  (Aust) | Osteoporosis and  Ultrasound Study (Europe) | Oxford  (UK) | Perth  (Aust) | Sheffield  (UK, McCloskey) | Sydney  (Aust) | Total |
| BMD affection status | High | 42 | 19 | 130 | 143 | 34 | 97 | 6 | 124 | 13 | 129 | 280 | 38 | 1055 |
|  | Low | 32 | 2 | 52 | 315 | 12 | 35 | 5 | 107 | 18 | 105 | 196 | 21 | 900 |
| Total participants with BMD |  | 74 | 21 | 182 | 458 | 46 | 132 | 11 | 231 | 31 | 234 | 476 | 59 | 1955 |
| Osteoporotic Fracture | Yes | 14 | 6 | 30 | 120 | 4 | 20 | 2 | 52 | 0 | 46 | 134 | 3 | 431 |
|  | No | 47 | 13 | 146 | 284 | 42 | 85 | 6 | 135 | 0 | 158 | 261 | 47 | 1224 |
|  | Unknown | 13 | 2 | 6 | 54 | 0 | 27 | 3 | 44 | 31 | 30 | 81 | 9 | 300 |
| Nonvertebral Osteoporotic Fracture | Yes | 14 | 5 | 20 | 94 | 3 | 12 | 2 | 20 | 0 | 32 | 89 | 3 | 294 |
|  | No | 47 | 13 | 146 | 282 | 42 | 85 | 6 | 137 | 0 | 158 | 261 | 47 | 1224 |
|  | Unknown | 13 | 3 | 16 | 82 | 1 | 35 | 3 | 74 | 31 | 44 | 126 | 9 | 437 |
| Hip Fracture | Yes | 2 | 1 | 0 | 19 | 0 | 0 | 1 | 1 | 0 | 3 | 29 | 0 | 56 |
|  | No | 72 | 20 | 182 | 434 | 46 | 127 | 10 | 230 | 0 | 227 | 447 | 56 | 1851 |
|  | Unknown | 0 | 0 | 0 | 5 | 0 | 5 | 0 | 0 | 31 | 4 | 0 | 3 | 48 |
| Vertebral Fracture | Yes | 0 | 2 | 18 | 23 | 0 | 11 | 0 | 42 | 0 | 21 | 69 | 0 | 186 |
|  | No | 0 | 0 | 0 | 0 | 0 | 0 | 0 | 183 | 0 | 0 | 404 | 0 | 587 |
|  | Unscreened | 74 | 19 | 164 | 435 | 46 | 121 | 11 | 6 | 31 | 213 | 3 | 59 | 1182 |
| Total per centre |  | 74 | 21 | 182 | 458 | 46 | 132 | 11 | 231 | 31 | 234 | 476 | 59 | 1955 |
