# Supplementary material for: Genome-Wide Association Study Using Extreme Truncate Selection Identifies Novel Genes Affecting Bone Mineral Density and Fracture Risk
Source: PLoS Genet. 2011 Apr 21;7(4):e1001372. doi: 10.1371/journal.pgen.1001372 (PMC3080863; doi:10.1371/journal.pgen.1001372)
Supplement: Table S2 — Descriptive statistics for discovery cohort. (0.06 MB DOC) [file pgen.1001372.s005.doc]

| CENTRE | BMD affection status | Number | Weight (kg) | Height (cm) | Age (years) at time of scan | z score total hip | BMD total hip in g/cm2 |
| --- | --- | --- | --- | --- | --- | --- | --- |
| Auckland (NZ) | High | 42 | 74 (13) | 160 (6.1) | 72 (6.2) | 1.9 (0.37) | 1.14 (0.06) |
|  | Low | 32 | 57 (7.8) | 160 (5.7) | 70 (6.5) | -2.0 (0.25) | 0.65 (0.06) |
| Brisbane (Aust) | High | 19 | 82 (15) | 163 (5.4) | 66 (6.7) | 2.1 (0.50) | 1.16 (0.11) |
|  | Low | 2 | 51 (6.9) | 156 (7.8) | 63 (4.2) | -1.7 (0.21) | 0.69 (0.11) |
| Dubbo (Aust) | High | 130 | 72 (14) | 160 (5.7) | 69 (5.9) | 2.0 (0.52) | 1.09 (0.10) |
|  | Low | 52 | 62 (13) | 157 (6.9) | 72 (6.5) | -1.8 (0.23) | 0.59 (0.06) |
| Geelong (Aust) | High | 143 | 72 (16) | 162 (6.2) | 67 (8.5) | 2.0 (0.45) | 1.13 (0.13) |
|  | Low | 315 | 70 (15) | 162 (7.6) | 65 (7.2) | -1.9 (0.36) | 0.73 (0.10) |
| Hertfordshire (UK) | High | 34 | 83 (17) | 162 (6.4) | 67 (2.6) | 2.1 (0.54) | 1.14 (0.07) |
|  | Low | 12 | 62 (11) | 157 (5.8) | 66 (2.4) | -2.0 (0.51) | 0.61 (0.07) |
| Hobart (Aust) | High | 97 | 80 (13) | 161 (6.1) | 68 (6.6) | 2.1 (0.57) | 1.13 (0.09) |
|  | Low | 35 | 58 (11) | 161 (7.0) | 62 (6.2) | -1.8 (0.27) | 0.65 (0.05) |
| Melbourne (Aust) | High | 6 | 72 (2.5) | 168 (5.6) | 64 (2.1) | 2.2 (0.29) | 1.11 (0.03) |
|  | Low | 5 | 59 (6.0) | 156 (6.4) | 60 (4.8) | -1.6 (0.12) | 0.66 (0.03) |
| Osteoporosis and Ultrasound Study (Europe) | High | 124 | 82 (13) | 161 (6.4) | 69 (5.9) | 2.0 (0.53) | 1.13 (0.09) |
|  | Low | 107 | 58 (8.2) | 158 (6.2) | 69 (6.6) | -1.9 (0.46) | 0.63 (0.08) |
| Oxford (UK) | High | 13 | 66 (6.7) | 162 (6.7) | 58 (7.8) | 2.4 (0.69) | 1.12 (0.16) |
|  | Low | 18 | 57 (8.8) | 157 (6.8) | 64 (7.7) | -2.1 (0.46) | 0.56 (0.09) |
| Perth (Aust) | High | 129 | 85 (15) | 163 (5.4) | 67 (6.5) | 2.1 (0.55) | 1.13 (0.09) |
|  | Low | 105 | 57 (8.6) | 158 (7.8) | 63 (6.0) | -1.8 (0.38) | 0.65 (0.06) |
| Sheffield (UK, McCloskey) | High | 280 | 78 (13) | 158 (5.9) | 79 (2.9) | 2.0 (0.44) | 1.03 (0.06) |
|  | Low | 196 | 55 (10) | 155 (6.3) | 79 (3.0) | -2.0 (0.44) | 0.51 (0.06) |
| Sydney (Aust) | High | 38 | 81 (16) | 162 (4.6) | 57 (5.9) | 2.0 (0.49) | 1.17 (0.08) |
|  | Low | 21 | 56 (9.0) | 159 (7.2) | 57 (7.5) | -1.9 (0.29) | 0.67 (0.05) |
