# Supplementary material for: Genome-Wide Association Study Using Extreme Truncate Selection Identifies Novel Genes Affecting Bone Mineral Density and Fracture Risk
Source: PLoS Genet. 2011 Apr 21;7(4):e1001372. doi: 10.1371/journal.pgen.1001372 (PMC3080863; doi:10.1371/journal.pgen.1001372)
Supplement: Table S3 — Replication cohort details. (0.04 MB DOC) [file pgen.1001372.s006.doc]

|  | Numbers | Weight (kg) | Height (cm) | Age (years) at time of BMD scan | Z score total hip | Mean BMD at total hip (g/cm^2^) |
| --- | --- | --- | --- | --- | --- | --- |
| Calcium Intake Fracture Outcome Study (Aust) | 322 | 68 (13) | 158 (6.3) | 75 (2.7) | 0.05(0.94) | 0.81 (0.12) |
| Dubbo Osteoporosis Epidemiology Study (Aust) | 818 | 69 (13) | 159 (6.2) | 73 (7.6) | -0.25 (0.98) | 0.84 (0.12) |
| Geelong Osteoporosis Study (Aust) | 1396 | 69 (14) | 160 (6.9) | 56 (19) | -0.1 (0.99) | 0.94 (0.17) |
| Oxford (UK) | 66 | 63 (10) | 160 (7.5) | 56 (9.9) | -0.9 (1.1) | 0.79 (0.14) |
| Osteoporosis and Ultrasound Study (Europe) | 1518 | 69 (11) | 160 (6.2) | 67 (7.2) | -0.02(0.68) | 0.88 (0.11) |
| Royal North Shore Hospital Twins Study (Aust) | 334 | 68 (12) | 161 (6.4) | 58 (8.8) | -0.10 (0.79) | 0.89 (0.11) |
| Sheffield (UK, McCloskey) | 4143 | 65 (11) | 156 (6.0) | 80 (4.0) | -0.03 (0.68) | 0.76 (0.11) |
| Tasmanian Older Adult Cohort (Aust) | 331 | 71 (13) | 161 (5.9) | 62 (7.2) | 0.2 (1.0) | 0.91 (0.12) |
