# Supplementary material for: Genome-Wide Association Study Using Extreme Truncate Selection Identifies Novel Genes Affecting Bone Mineral Density and Fracture Risk
Source: PLoS Genet. 2011 Apr 21;7(4):e1001372. doi: 10.1371/journal.pgen.1001372 (PMC3080863; doi:10.1371/journal.pgen.1001372)
Supplement: Table S4 — Replication cohort fracture data. (0.04 MB DOC) [file pgen.1001372.s007.doc]

|  | | CENTRE | | | | | | | |  |
| --- | --- | --- | --- | --- | --- | --- | --- | --- | --- | --- |
|  |  | Calcium Intake Fracture Outcome Study (Aust) | Dubbo Osteoporosis Epidemiology Study (Aust) | Geelong Osteoporosis Study (Aust) | Osteoporosis and Ultrasound Study (Europe) | Oxford (UK) | Royal North Shore Hospital Twins Study (Aust) | Sheffield  (UK, McCloskey) | Tasmanian Older Adult Cohort (Aust) | Total |
| Osteoporotic Fracture | Yes | 35 | 267 | 239 | 256 | 0 | 19 | 1408 | 129 | 2353 |
|  | No | 0 | 484 | 1097 | 1034 | 0 | 294 | 2082 | 155 | 5146 |
|  | Unknown | 287 | 67 | 60 | 228 | 66 | 21 | 653 | 47 | 1429 |
| Nonvertebral Osteoporotic Fracture | Yes | 20 | 175 | 167 | 99 | 0 | 18 | 980 | 28 | 1487 |
|  | No | 0 | 484 | 1097 | 1039 | 0 | 294 | 2099 | 156 | 5169 |
|  | Unknown | 302 | 159 | 132 | 380 | 66 | 22 | 1064 | 147 | 2272 |
| Hip Fracture | Yes | 20 | 7 | 47 | 13 | 0 | 0 | 192 | 3 | 282 |
|  | No | 0 | 811 | 1349 | 1505 | 0 | 334 | 3951 | 327 | 8277 |
|  | Unknown | 302 | 0 | 0 | 0 | 66 | 0 | 0 | 1 | 369 |
| Vertebral Fracture | Yes | 15 | 140 | 76 | 191 | 0 | 7 | 581 | 112 | 1122 |
|  | No | 0 | 0 | 0 | 1298 | 0 | 0 | 3522 | 195 | 5015 |
|  | Unscreened | 307 | 678 | 1320 | 29 | 66 | 327 | 40 | 24 | 2791 |
| Total persons per centre | | 322 | 818 | 1396 | 1518 | 66 | 334 | 4143 | 331 | 8928 |
