# Supplementary material for: Genome-Wide Association Study Using Extreme Truncate Selection Identifies Novel Genes Affecting Bone Mineral Density and Fracture Risk
Source: PLoS Genet. 2011 Apr 21;7(4):e1001372. doi: 10.1371/journal.pgen.1001372 (PMC3080863; doi:10.1371/journal.pgen.1001372)
Supplement: Table S5 — Replication study SNPs, beta coefficients and P-values for analysis of TH, FN and LS. The regression coefficient in the case-control analysis of TH in the discovery set shows the expected increase in the log odds ratio of low BMD per addition of allele A2. The regression coefficients in the TH, FN and LS analyses refer to the expected increase in standardized BMD per addition of allele A2 in the discovery set. (0.22 MB DOC) [file pgen.1001372.s008.doc]

|  |  |  |  | Genotyping method | REASON FOR INCLUSION | Discovery set  case-control (TH) | | TH | | FN | | LS | |
| --- | --- | --- | --- | --- | --- | --- | --- | --- | --- | --- | --- | --- | --- |
| MARKER | Chrom. | Position | Alleles  A1/A2 |  |  | Beta | P-value | Beta | P-value | Beta | P-value | Beta | P-value |
| rs10915311 | 1 | 4968815 | C/A | OpenArray | GRAIL | -0.17 | 0.0097 | 0.056 | 0.0075 | 0.055 | 0.015 | 0.045 | 0.18 |
| rs7536712 | 1 | 5153524 | T/C | OpenArray | P-VALUE | 0.22 | 0.00089 | -0.074 | 0.00042 | -0.061 | 0.0070 | -0.048 | 0.15 |
| rs4920332 | 1 | 18704842 | T/C | OpenArray | P-VALUE | -0.16 | 0.034 | 0.044 | 0.026 | 0.037 | 0.086 | 0.064 | 0.043 |
| rs7524102 | 1 | 22571034 | G/A | OpenArray | GRAIL | 0.44 | 8.7x10^-7^ | -0.14 | 1.0x10^-6^ | -0.14 | 1.3x10^-6^ | -0.13 | 0.0026 |
| rs6700352 | 1 | 41844025 | G/T | OpenArray | P-VALUE | 0.22 | 0.019 | -0.058 | 0.042 | -0.061 | 0.045 | -0.01 | 0.77 |
| rs3911861 | 1 | 44835040 | C/T | OpenArray | P-VALUE | 0.19 | 0.0076 | -0.060 | 0.0052 | -0.042 | 0.069 | -0.048 | 0.16 |
| rs7550034 | 11 | 92137164 | G/A | Taqman | P-VALUE | 0.26 | 6.7x10^-5^ | -0.080 | 1.5x10^-4^ | -0.07 | 0.001 | -0.032 | 0.35 |
| rs12566180 | 1 | 92135390 | C/T | Taqman | P-VALUE | 0.26 | 9.5x10^-5^ | -0.075 | 3.1x10^-4^ | -0.07 | 9.5x10^-4^ | -0.031 | 0.36 |
| rs10494257 | 1 | 145721193 | C/T | OpenArray | P-VALUE | -0.21 | 0.011 | 0.040 | 0.13 | 0.016 | 0.56 | 0.048 | 0.26 |
| rs1353428 | 1 | 145792846 | C/A | OpenArray | P-VALUE | -0.23 | 0.00089 | 0.053 | 0.013 | 0.036 | 0.11 | 0.028 | 0.42 |
| rs2872977 | 2 | 4975854 | G/A | OpenArray | P-VALUE | 0.37 | 0.0020 | -0.077 | 0.031 | -0.072 | 0.061 | -0.098 | 0.095 |
| rs979290 | 2 | 7599822 | A/G | OpenArray | P-VALUE | 0.21 | 0.0021 | -0.035 | 0.12 | -0.04 | 0.047 | 0.023 | 0.51 |
| rs957870 | 2 | 76832923 | G/T | OpenArray | P-VALUE | -0.22 | 0.00080 | 0.063 | 0.0027 | 0.055 | 0.016 | 0.079 | 0.018 |
| rs289871 | 2 | 151773642 | A/G | OpenArray | P-VALUE | -0.31 | 1.8x10^-5^ | 0.064 | 0.00062 | 0.060 | 0.0029 | 0.021 | 0.48 |
| rs777355 | 2 | 166262283 | A/G | OpenArray | GRAIL | -0.27 | 5.6x10^-5^ | 0.075 | 0.00026 | 0.084 | 0.00013 | 0.057 | 0.081 |
| rs6710518 | 2 | 166291490 | T/C | Taqman | P-VALUE | -0.26 | 6.9x10^-5^ | 0.078 | 0.00015 | 0.091 | 3.9x10^-5^ | 0.068 | 0.038 |
| rs4667492 | 2 | 166295948 | C/T | OpenArray | P-VALUE | 0.25 | 0.00081 | -0.075 | 0.0014 | -0.10 | 4.4x10^-5^ | -0.052 | 0.16 |
| rs10930846 | 2 | 179548078 | G/A | OpenArray | P-VALUE | -0.25 | 0.0013 | 0.045 | 0.042 | 0.020 | 0.40 | 0.045 | 0.20 |
| rs1866852 | 2 | 190949431 | C/T | OpenArray | P-VALUE | -0.27 | 5.2x10^-5^ | 0.086 | 4.6x10^-5^ | 0.077 | 0.00062 | 0.072 | 0.033 |
| rs2541155 | 2 | 205903478 | C/A | OpenArray | GRAIL | -0.26 | 0.0032 | 0.068 | 0.019 | 0.048 | 0.12 | 0.0029 | 0.95 |
| rs2372866 | 2 | 217294227 | T/C | OpenArray | GRAIL | 0.31 | 0.0097 | -0.060 | 0.12 | -0.023 | 0.57 | -0.076 | 0.21 |
| rs12623903 | 2 | 217781637 | C/T | OpenArray | P-VALUE | 0.31 | 0.00054 | -0.095 | 0.00052 | -0.096 | 0.0012 | -0.14 | 0.0013 |
| rs1827452 | 3 | 2395109 | A/G | OpenArray | P-VALUE | 0.45 | 0.00064 | -0.12 | 0.0027 | -0.11 | 0.012 | -0.17 | 0.0096 |
| rs17595878 | 3 | 3002090 | A/G | OpenArray | P-VALUE | 0.53 | 0.0022 | -0.18 | 0.00073 | -0.17 | 0.0040 | -0.35 | 8.1x10^-5^ |
| rs10510311 | 3 | 5491427 | A/G | OpenArray | P-VALUE | -0.50 | 0.00049 | 0.14 | 0.0016 | 0.14 | 0.0045 | 0.18 | 0.012 |
| rs13079656 | 3 | 18068084 | G/A | OpenArray | P-VALUE | 0.37 | 0.0021 | -0.091 | 0.016 | -0.063 | 0.12 | -0.089 | 0.15 |
| rs6767354 | 3 | 23759307 | A/G | OpenArray | P-VALUE | -0.31 | 0.00019 | 0.078 | 0.0032 | 0.096 | 0.00075 | 0.073 | 0.082 |
| rs10513437 | 3 | 154140815 | T/C | OpenArray | P-VALUE | 0.26 | 0.002 | -0.091 | 0.00069 | -0.075 | 0.0089 | 2.0 x10^-5^ | 1.0 |
| rs6816987 | 4 | 43259588 | T/C | OpenArray | P-VALUE | -0.38 | 0.0010 | 0.12 | 0.0017 | 0.11 | 0.0090 | 0.050 | 0.40 |
| rs6447290 | 4 | 43338432 | A/G | OpenArray | P-VALUE | -0.35 | 0.00086 | 0.11 | 0.0012 | 0.095 | 0.0072 | 0.075 | 0.15 |
| rs2085292 | 4 | 48609693 | G/A | OpenArray | GRAIL | -0.22 | 0.00073 | 0.059 | 0.0049 | 0.068 | 0.0025 | 0.12 | 0.00046 |
| rs11934856 | 4 | 85658026 | A/G | OpenArray | GRAIL | -0.26 | 0.00035 | 0.062 | 0.0038 | 0.048 | 0.037 | 0.048 | 0.18 |
| rs1838039 | 4 | 85844725 | A/G | OpenArray | P-VALUE | 0.30 | 1.4x10^-5^ | -0.065 | 0.0023 | -0.051 | 0.026 | -0.037 | 0.29 |
| rs1462367 | 4 | 88789552 | C/T | OpenArray | GRAIL | -0.28 | 0.00013 | 0.081 | 0.00060 | 0.083 | 0.0011 | 0.036 | 0.35 |
| rs2732171 | 4 | 88875008 | A/G | OpenArray | GRAIL | -0.24 | 0.0011 | 0.071 | 0.0028 | 0.070 | 0.0064 | 0.029 | 0.45 |
| rs1054627 | 4 | 88951716 | A/G | OpenArray | GRAIL | -0.28 | 0.000066 | 0.071 | 0.0015 | 0.079 | 0.00095 | 0.048 | 0.18 |
| rs419764 | 4 | 108063606 | T/C | OpenArray | GRAIL | 0.29 | 0.000035 | -0.076 | 0.00070 | -0.061 | 0.012 | -0.029 | 0.42 |
| rs6849420 | 4 | 153309892 | T/C | OpenArray | P-VALUE | 0.23 | 0.0041 | -0.063 | 0.014 | -0.063 | 0.020 | -0.047 | 0.25 |
| rs10517644 | 4 | 157716553 | A/G | OpenArray | GRAIL | -0.24 | 0.022 | 0.046 | 0.16 | 0.047 | 0.18 | -0.033 | 0.53 |
| rs1020993 | 4 | 176210389 | T/C | OpenArray | P-VALUE | 0.29 | 0.022 | -0.042 | 0.043 | -0.038 | 0.084 | -0.071 | 0.029 |
| rs293123 | 5 | 4697270 | G/A | OpenArray | P-VALUE | -0.15 | 0.019 | 0.058 | 0.0064 | 0.075 | 0.00091 | 0.084 | 0.013 |
| rs2561858 | 5 | 5912422 | A/G | OpenArray | P-VALUE | -0.24 | 0.00036 | 0.065 | 0.0033 | 0.063 | 0.0076 | 0.023 | 0.51 |
| rs888786 | 5 | 75006606 | A/G | OpenArray | P-VALUE | -0.28 | 0.0026 | 0.068 | 0.025 | 0.043 | 0.19 | 0.048 | 0.32 |
| rs6868762 | 5 | 80548730 | T/G | OpenArray | P-VALUE | -0.22 | 0.00088 | 0.070 | 0.0013 | 0.070 | 0.0027 | 0.081 | 0.02 |
| rs750728 | 5 | 80565512 | T/C | OpenArray | P-VALUE | -0.26 | 0.00034 | 0.074 | 0.0017 | 0.089 | 0.00046 | 0.10 | 0.0065 |
| rs10074794 | 5 | 80617207 | A/G | OpenArray | P-VALUE | -0.25 | 0.00080 | 0.071 | 0.0027 | 0.083 | 0.0011 | 0.093 | 0.015 |
| rs4957798 | 5 | 108472453 | T/C | OpenArray | P-VALUE | 0.15 | 0.069 | -0.051 | 0.049 | -0.072 | 0.0099 | -0.040 | 0.33 |
| rs13182913 | 5 | 147975798 | C/T | OpenArray | GRAIL | -0.25 | 0.00023 | 0.067 | 0.0027 | 0.054 | 0.025 | 0.042 | 0.25 |
| rs7728260 | 5 | 174361691 | C/T | OpenArray | P-VALUE | -0.27 | 0.00068 | 0.070 | 0.0061 | 0.070 | 0.012 | 0.013 | 0.76 |
| rs12524808 | 6 | 1133835 | A/G | OpenArray | P-VALUE | -0.35 | 0.021 | 0.077 | 0.11 | 0.078 | 0.13 | -0.034 | 0.66 |
| rs1029124 | 6 | 10287352 | T/C | OpenArray | GRAIL | -0.41 | 0.0050 | 0.078 | 0.083 | 0.082 | 0.091 | 0.078 | 0.29 |
| rs4712613 | 6 | 21486134 | G/T | Taqman | P-VALUE | -0.22 | 0.00093 | -0.078 | 0.00030 | -0.089 | 0.00012 | -0.096 | 0.0054 |
| rs9466056 | 6 | 21492592 | A/G | Taqman | P-VALUE | -0.24 | 0.00053 | 0.079 | 0.00020 | 0.090 | 6.6 x10^-5^ | 0.099 | 0.0036 |
| rs6915083 | 6 | 41271983 | G/A | OpenArray | GRAIL | -0.27 | 7.7x10^-5^ | 0.067 | 0.0019 | 0.066 | 0.0042 | 0.082 | 0.017 |
| rs10944237 | 6 | 87282208 | T/G | OpenArray | GRAIL | 0.23 | 0.0041 | -0.057 | 0.030 | -0.060 | 0.031 | -0.0091 | 0.83 |
| rs10498988 | 6 | 94568251 | C/T | OpenArray | P-VALUE | 0.44 | 0.0028 | -0.11 | 0.015 | -0.049 | 0.32 | 0.047 | 0.51 |
| rs4240565 | 6 | 99274243 | C/T | OpenArray | P-VALUE | 0.44 | 0.0013 | -0.14 | 0.0012 | -0.14 | 0.0019 | -0.040 | 0.55 |
| rs3734678 | 6 | 107639853 | G/T | OpenArray | P-VALUE | 0.23 | 0.0080 | -0.063 | 0.020 | -0.071 | 0.016 | -0.078 | 0.077 |
| rs9386654 | 6 | 108039974 | A/G | OpenArray | P-VALUE | 0.42 | 3.3x10^-5^ | -0.13 | 1.8x10^-5^ | -0.13 | 0.00012 | -0.10 | 0.038 |
| rs9372313 | 6 | 112248783 | T/C | OpenArray | P-VALUE | -0.51 | 0.0051 | 0.093 | 0.10 | 0.12 | 0.049 | 0.13 | 0.17 |
| rs13204965 | 6 | 127206265 | C/A | Taqman | P-VALUE | -0.3 | 0.00021 | -0.033 | 0.023 | -0.041 | 0.012 | -0.053 | 0.045 |
| rs17563605 | 6 | 127208765 | C/T | Taqman | P-VALUE | -0.3 | 0.00021 | 0.031 | 0.027 | 0.032 | 0.043 | 0.050 | 0.057 |
| rs1406937 | 6 | 132617607 | T/C | OpenArray | P-VALUE | -0.48 | 0.0013 | 0.098 | 0.0066 | 0.081 | 0.037 | 0.070 | 0.22 |
| rs2529015 | 7 | 20293111 | T/C | OpenArray | GRAIL | 0.25 | 0.0055 | -0.065 | 0.018 | -0.082 | 0.0056 | -0.078 | 0.084 |
| rs2462131 | 7 | 79129442 | C/T | OpenArray | P-VALUE | 0.23 | 0.00055 | -0.064 | 0.0022 | -0.057 | 0.012 | -0.058 | 0.081 |
| rs4342521 | 7 | 95973941 | T/G | Taqman | P-VALUE | -0.25 | 0.00061 | 0.070 | 0.00088 | 0.078 | 0.00059 | 0.11 | 0.0016 |
| rs11974704 | 7 | 95980680 | A/G | Taqman | P-VALUE | 6.828 | 0.073 | -0.18 | 0.038 | -0.086 | 0.35 | -0.59 | 6.7x10^-6^ |
| rs1528353 | 7 | 120953045 | A/G | OpenArray | GRAIL | 0.20 | 0.0023 | -0.059 | 0.0046 | -0.069 | 0.0020 | -0.066 | 0.049 |
| rs2214706 | 7 | 121011093 | T/C | OpenArray | GRAIL | 0.18 | 0.0084 | -0.056 | 0.0077 | -0.066 | 0.0030 | -0.083 | 0.014 |
| rs960840 | 7 | 152692346 | G/A | OpenArray | P-VALUE | -0.15 | 0.023 | 0.033 | 0.13 | 0.042 | 0.071 | 0.050 | 0.15 |
| rs623202 | 8 | 2821201 | G/A | OpenArray | P-VALUE | 0.3 | 0.0010 | -0.049 | 0.073 | -0.045 | 0.12 | -0.016 | 0.72 |
| rs11203505 | 8 | 13549817 | C/A | OpenArray | GRAIL | 0.36 | 0.0013 | -0.099 | 0.0057 | -0.087 | 0.024 | -0.11 | 0.067 |
| rs3848988 | 8 | 13631654 | T/C | OpenArray | GRAIL | 0.28 | 0.0031 | -0.082 | 0.0071 | -0.091 | 0.0053 | -0.084 | 0.084 |
| rs17497685 | 8 | 21287480 | T/C | OpenArray | P-VALUE | 0.34 | 0.0038 | -0.10 | 0.0052 | -0.14 | 0.00046 | -0.12 | 0.031 |
| rs2467691 | 8 | 25616924 | G/A | OpenArray | GRAIL | -0.17 | 0.047 | 0.024 | 0.41 | 0.023 | 0.47 | 0.028 | 0.56 |
| rs1481513 | 8 | 79171163 | G/A | OpenArray | GRAIL | -0.20 | 0.0024 | 0.061 | 0.0043 | 0.064 | 0.0051 | 0.048 | 0.16 |
| rs1529989 | 8 | 85306377 | C/A | OpenArray | P-VALUE | -0.20 | 0.0027 | 0.048 | 0.018 | 0.046 | 0.036 | 0.039 | 0.25 |
| rs6990827 | 8 | 130448512 | G/T | OpenArray | P-VALUE | 0.23 | 0.00047 | 0.075 | 0.00037 | 0.074 | 0.0012 | 0.092 | 0.0063 |
| rs2703004 | 8 | 134153982 | G/A | OpenArray | GRAIL | 0.23 | 0.00038 | -0.083 | 9.3x10^-5^ | -0.086 | 0.00017 | -0.081 | 0.017 |
| rs2997668 | 9 | 71869498 | G/A | OpenArray | P-VALUE | -0.46 | 0.00022 | 0.125 | 0.0013 | 0.15 | 0.00032 | 0.13 | 0.042 |
| rs13285182 | 9 | 74642380 | A/G | OpenArray | P-VALUE | -0.28 | 0.0012 | 0.082 | 0.0034 | 0.074 | 0.013 | -0.011 | 0.81 |
| rs4745209 | 9 | 74829925 | C/T | OpenArray | P-VALUE | -0.19 | 0.011 | 0.058 | 0.015 | 0.070 | 0.0065 | 0.029 | 0.44 |
| rs7021801 | 9 | 119125525 | C/A | OpenArray | P-VALUE | 0.19 | 0.0033 | -0.073 | 0.00063 | -0.052 | 0.023 | -0.093 | 0.0070 |
| rs4074715 | 10 | 68226404 | G/A | OpenArray | GRAIL | 0.45 | 0.0011 | -0.13 | 0.0024 | -0.15 | 0.0013 | -0.039 | 0.57 |
| rs3847346 | 10 | 71123154 | C/A | OpenArray | GRAIL | -0.23 | 0.0062 | 0.037 | 0.036 | 0.038 | 0.046 | 0.027 | 0.33 |
| rs2420936 | 10 | 123198871 | A/G | OpenArray | GRAIL | 0.22 | 0.00096 | -0.058 | 0.0051 | -0.061 | 0.0069 | -0.040 | 0.23 |
| rs13448 | 11 | 46651990 | C/T | OpenArray | GRAIL | 0.28 | 0.00079 | -0.057 | 0.015 | -0.053 | 0.038 | -0.072 | 0.057 |
| rs6485690 | 11 | 46755207 | A/G | OpenArray | GRAIL | 0.17 | 0.016 | -0.035 | 0.12 | -0.032 | 0.18 | -0.051 | 0.15 |
| rs6485702 | 11 | 46855347 | T/C | OpenArray | GRAIL | 0.17 | 0.013 | -0.036 | 0.10 | -0.033 | 0.17 | -0.051 | 0.15 |
| rs1152619 | 11 | 65012165 | A/C | Taqman | P-VALUE | 0.27 | 0.00026 | -0.03 | 0.038 | 0.016 | 0.32 | 0.0011 | 0.97 |
| rs1152620 | 11 | 65013905 | G/A | OpenArray | GRAIL | 0.31 | 4.4x10^-5^ | -0.079 | 0.00099 | -0.060 | 0.020 | -0.080 | 0.041 |
| rs12797615 | 11 | 85944331 | C/T | OpenArray | P-VALUE | 0.25 | 0.00056 | -0.057 | 0.011 | -0.061 | 0.012 | -0.057 | 0.12 |
| rs1374475 | 11 | 92605285 | C/T | OpenArray | P-VALUE | -0.32 | 7.3x10^-5^ | 0.092 | 0.00042 | 0.093 | 0.00087 | 0.082 | 0.050 |
| rs518181 | 11 | 116277997 | C/A | OpenArray | GRAIL | -0.14 | 0.029 | 0.039 | 0.080 | 0.011 | 0.64 | 0.0080 | 0.83 |
| rs10876279 | 12 | 51012062 | T/C | OpenArray | P-VALUE | 0.28 | 0.00070 | -0.064 | 0.018 | -0.069 | 0.016 | -0.10 | 0.018 |
| rs11106105 | 12 | 90268652 | T/G | OpenArray | GRAIL | 0.21 | 0.0049 | -0.071 | 0.0028 | -0.072 | 0.0047 | -0.15 | 8.5x10^-5^ |
| rs11106110 | 12 | 90277126 | G/T | OpenArray | GRAIL | 0.21 | 0.0052 | -0.071 | 0.0030 | -0.071 | 0.0051 | -0.15 | 8.5x10^-5^ |
| rs7328533 | 13 | 39315048 | T/C | OpenArray | P-VALUE | -0.58 | 1.0x10^-5^ | 0.15 | 0.0027 | 0.14 | 0.0070 | 0.20 | 0.0077 |
| rs10507508 | 13 | 41867782 | G/A | OpenArray | GRAIL | 0.57 | 0.00017 | -0.21 | 1.4x10^-5^ | -0.18 | 0.00029 | -0.21 | 0.0044 |
| rs1928042 | 13 | 46335217 | G/T | OpenArray | GRAIL | 0.17 | 0.029 | -0.045 | 0.052 | -0.047 | 0.057 | 0.0021 | 0.96 |
| rs1414011 | 13 | 87001078 | C/T | OpenArray | GRAIL | -0.31 | 0.0029 | 0.069 | 0.044 | 0.073 | 0.047 | 0.097 | 0.076 |
| rs959536 | 13 | 87069297 | T/C | OpenArray | GRAIL | -0.32 | 0.0023 | 0.070 | 0.039 | 0.074 | 0.042 | 0.10 | 0.064 |
| rs529060 | 13 | 96021571 | C/T | OpenArray | P-VALUE | 0.30 | 0.00028 | -0.068 | 0.0093 | -0.058 | 0.040 | -0.081 | 0.059 |
| rs12915209 | 15 | 43827749 | T/G | OpenArray | P-VALUE | -0.19 | 0.0071 | 0.066 | 0.0035 | 0.049 | 0.046 | 0.024 | 0.50 |
| rs13336428 | 16 | 1472464 | A/G | OpenArray | P-VALUE | -0.22 | 0.00070 | 0.065 | 0.0026 | 0.057 | 0.013 | 0.076 | 0.028 |
| rs1004299 | 16 | 52901341 | T/C | OpenArray | GRAIL | -0.17 | 0.012 | 0.056 | 0.0085 | 0.060 | 0.0084 | 0.11 | 0.00093 |
| rs731258 | 16 | 82271433 | T/C | OpenArray | P-VALUE | -0.22 | 0.0069 | 0.057 | 0.023 | 0.072 | 0.0077 | 0.046 | 0.24 |
| rs4843966 | 16 | 85074601 | G/T | OpenArray | P-VALUE | -0.40 | 0.00016 | 0.13 | 8.1x10^-5^ | 0.16 | 1.3x10^-5^ | 0.039 | 0.45 |
| rs205060 | 17 | 11233236 | T/C | OpenArray | P-VALUE | -0.22 | 0.0010 | 0.048 | 0.022 | 0.043 | 0.058 | 0.032 | 0.35 |
| rs3744811 | 18 | 13639542 | T/C | OpenArray | GRAIL | -0.24 | 0.0017 | 0.070 | 0.0039 | 0.065 | 0.013 | 0.039 | 0.31 |
| rs8098464 | 18 | 28516811 | G/A | OpenArray | P-VALUE | -0.48 | 0.00027 | 0.081 | 0.0081 | 0.070 | 0.034 | 0.038 | 0.45 |
| rs4486998 | 18 | 36923888 | T/G | OpenArray | P-VALUE | 0.41 | 6.6x10^-6^ | -0.069 | 0.016 | -0.095 | 0.0020 | -0.058 | 0.21 |
| rs1470113 | 18 | 39563514 | T/C | OpenArray | P-VALUE | 0.23 | 0.0022 | -0.082 | 0.00042 | -0.098 | 8.4 x10^-5^ | -0.051 | 0.17 |
| rs2543025 | 18 | 41403974 | C/T | OpenArray | P-VALUE | 0.25 | 0.00011 | -0.065 | 0.0020 | -0.076 | 0.00083 | -0.065 | 0.054 |
| rs984429 | 18 | 47941339 | T/G | OpenArray | P-VALUE | -0.27 | 0.0015 | 0.052 | 0.051 | 0.057 | 0.047 | 0.044 | 0.31 |
| rs10514112 | 18 | 69957348 | C/T | OpenArray | P-VALUE | 0.28 | 0.0088 | -0.086 | 0.0097 | -0.097 | 0.0064 | -0.15 | 0.0062 |
| rs7253057 | 19 | 53172856 | T/C | OpenArray | GRAIL | 0.18 | 0.0097 | -0.056 | 0.013 | -0.037 | 0.13 | -0.0038 | 0.92 |
| rs3219433 | 19 | 556110781 | G/A | Taqman | P-VALUE | 0.43 | 0.0013 | 0.097 | 0.017 | 0.082 | 0.061 | 0.089 | 0.17 |
| rs6036158 | 20 | 22529060 | C/T | OpenArray | GRAIL | -0.39 | 0.0065 | 0.12 | 0.0062 | 0.12 | 0.014 | 0.11 | 0.13 |
| rs6022676 | 20 | 51744861 | C/A | OpenArray | P-VALUE | -0.27 | 0.00085 | 0.076 | 9.1x10^-5^ | 0.077 | 0.00021 | 0.071 | 0.023 |
| rs290403 | 20 | 52144921 | A/G | OpenArray | P-VALUE | 0.29 | 1.7x10^-5^ | -0.085 | 4.8x10^-5^ | -0.091 | 4.7 x10^-5^ | -0.087 | 0.0088 |
| rs3814902 | 21 | 42689617 | G/A | OpenArray | P-VALUE | 0.37 | 0.0018 | -0.077 | 0.021 | -0.099 | 0.0061 | -0.088 | 0.10 |
| rs3827380 | 22 | 38217632 | G/A | OpenArray | GRAIL | -0.25 | 0.0012 | 0.067 | 0.0091 | 0.051 | 0.065 | -0.021 | 0.62 |
| rs761917 | 22 | 46636957 | G/T | OpenArray | P-VALUE | 0.22 | 0.00065 | -0.084 | 9.7x10^-5^ | -0.080 | 0.00054 | 0.0032 | 0.93 |
