# Supplementary material for: Genome-Wide Association Study Using Extreme Truncate Selection Identifies Novel Genes Affecting Bone Mineral Density and Fracture Risk
Source: PLoS Genet. 2011 Apr 21;7(4):e1001372. doi: 10.1371/journal.pgen.1001372 (PMC3080863; doi:10.1371/journal.pgen.1001372)
Supplement: Table S6 — Association findings in AOGC discovery set for markers achieving genome-wide significant association with BMD in previous studies. The regression coefficient in the TH analysis shows the expected increase in the log odds ratio of low BMD per addition of allele A2. The regression coefficients in the FN and LS analyses refer to the expected increase in standardized BMD per addition of allele A2. (0.12 MB DOC) [file pgen.1001372.s009.doc]

|  |  |  | |  | AOGC DISCOVERY SET | | | | | | PREVIOUS REPORT | | | | | | | | |
| --- | --- | --- | --- | --- | --- | --- | --- | --- | --- | --- | --- | --- | --- | --- | --- | --- | --- | --- | --- |
|  |  |  | |  | TH | | FN | | LS | | LS | | FN | | Distal Radius | | Whole Body | |  |
| LOCUS | GENE | | SNP | A1/A2 | BETA | P-VALUE | BETA | P-VALUE | BETA | P-VALUE | BETA | P-VALUE | BETA | P-VALUE | BETA | P-VALUE | BETA | P-VALUE | STUDY |
| 1p31.3 | *GPR177* | | rs1430742 | C/T | 0.10 | 0.21 | 0.041 | 0.13 | 0.019 | 0.64 | 0.105 | 2.5x10^-11^ | 0.1 | 1.8x10^-12^ | - | - | - | - | GEFOS [17] |
|  |  | | rs2195682 | C/T | 0.266 | 7.1x10^-5^ | 0.070 | 0.0021 | 0.05 | 0.11 | - | - | - | - | - | - | - | - | AOGC |
| 1p36 | *ZBTB40* | | rs7524102 | A/G | -0.44 | 8.7x10^-7^ | -0.14 | 1.3x10^-6^ | -0.13 | 0.0026 | -0.11 | 9.2x10^-9^ | -0.15 | 5.0x10^-16^ | - | - | - | - | DECODE-1 [23] |
|  |  | | rs6696981 | G/T | -0.30 | 0.0038 | -0.11 | 0.0013 | -0.14 | 0.0087 | -0.12 | 1.2x10^-7^ | -0.14 | 3.8x10^-12^ | - | - | - | - | DECODE-1 [23] |
| 2p16 | *SBTBN1* | | rs11898505 | A/G | -0.018 | 0.79 | 0.0 | 0.71 | 0.052 | 0.14 | -0.08 | 8.4x10^-7^ | - | - | - | - | - | - | DECODE-1 [23] |
| 3p22 | *CTNNB1* | | rs87938 | A/G | -0.27 | 3.5x10^-5^ | -0.096 | 2.2x10^-5^ | -0.078 | 0.021 | -0.043 | 3.1x10^-4^ | -0.07 | 3.4x10^-9^ | - | - | - | - | GEFOS [17] |
| 4q21.1 | *MEPE* | | rs1471403 | C/T | -0.18 | 0.011 | -0.048 | 0.042 | -0.058 | 0.10 | -0.068 | 5.7x10^-8^ | -0.059 | 2.0x10^-6^ | - | - | - | - | GEFOS [17] |
| 5q14 | *MEF2C* | | rs1366594 | A/C | 0.35 | 1.3x10^-7^ | 0.14 | 8.0x10^-10^ | 0.071 | 0.029 | -0.005 | NS | 0.085 | 1.1x10^-12^ | - | - | - | - | GEFOS [17] |
| 6p21 | *MHC* | | rs3130340 | C/T | 0.15 | 0.061 | 0.02 | 0.43 | 0.0 | 0.95 | -0.10 | 1.2x10^-7^ | -0.05 | 0.007 | - | - | - | - | DECODE-1 [23] |
| 6q25 | *C6orf97/ESR1* | | rs9579055 | - | - | - | - | - | - | - | -0.08 | 6.2x10^-7^ | -0.08 | 3.1x10^-8^ | - | - | - | - | DECODE-1 [23] |
|  |  | | rs4870044 | C/T | 0.20 | 0.0074 | 0.061 | 0.012 | 0.061 | 0.085 | -0.11 | 1.6x10^-11^ | -0.08 | 1.6x10^-7^ | - | - | - | - | DECODE-1 [23] |
|  |  | | rs1038304 | A/G | 0.10 | 0.12 | 0.041 | 0.064 | 0.082 | 0.013 | -0.10 | 4.0x10^-11^ | -0.08 | 5.3x10^-9^ | - | - | - | - | DECODE-1 [23] |
|  |  | | rs6929137 | A/G | -0.032 | 0.64 | -0.026 | 0.27 | -0.059 | 0.091 | -0.10 | 2.5x10^-10^ | -0.08 | 1.0x10^-7^ | - | - | - | - | DECODE-1 [23] |
|  |  | | rs1999805 | A/G | 0.12 | 0.071 | 0.022 | 0.32 | 0.069 | 0.040 | -0.09 | 2.2x10^-8^ | -0.06 | 1.2x10^-4^ | - | - | - | - | DECODE-1 [23] |
| 7p14 | *STARD3NL* | | rs1524058 | C/T | 0.14 | 0.033 | 0.021 | 0.33 | 0.055 | 0.089 | 0.07 | 5.2x10^-9^ | 0.038 | 1.4x10^-3^ | - | - | - | - | GEFOS [17] |
| 7q21.3 | *FLJ42280* | | rs4729260 | C/G | 0.27 | 3.6x10^-4^ | 0.083 | 2.2x10^-4^ | 0.11 | 0.0017 | 0.081 | 9.5x10^-10^ | 0.085 | 5.4x10^-11^ | - | - | - | - | GEFOS [17] |
|  |  | | rs7781370 | C/T | 0.25 | 6.9x10^-4^ | 0.077 | 6.9x10^-4^ | 0.11 | 0.0020 | 0.074 | 5.5x10^-9^ | 0.083 | 2.9x10^-11^ | - | - | - | - | GEFOS [17] |
| 7q31 | *FAM3C* | | rs7776725 | C/T | 0.23 | 0.0031 | 0.063 | 0.0062 | 0.066 | 0.058 | - | - | - | - | 0.212 | 1.0x10^-11^ | - | - | CHO ET AL [32]. |
| 8q24 | *OPG* | | rs6993813 | C/T | -0.26 | 1.1x10^-4^ | -0.085 | 1.4x10^-4^ | -0.11 | 9.9x10^-4^ | -0.12 | 1.8x10^-14^ | -0.09 | 3.3x10^-11^ | - | - | - | - | DECODE-1 [23] |
|  |  | | rs6469804 | A/G | -0.25 | 1.9x10^-4^ | -0.074 | 9.3x10^-4^ | -0.10 | 0.0023 | -0.12 | 7.4x10^-15^ | -0.08 | 2.5x10^-9^ | - | - | - | - | DECODE-1 [23] |
|  |  | | rs4355801 | A/G | -0.26 | 8.5x10^-5^ | -0.075 | 0.0010 | -0.074 | 0.027 | 0.09 | 7.6x10^-10^ | - | - | - | - | - | - | TWINS UK / ROTTERDAM [21] |
| 11p14.1 | *DCDC5* | | rs16921914 | A/G | 0.10 | 0.17 | 0.011 | 0.62 | 0.029 | 0.43 | 0.077 | 1.0x10^-8^ | 0.013 | 0.005 | - | - | - | - | GEFOS [17] |
| 11p15 | *SOX6* | | rs7117858 | A/G | -0.30 | 1.9x10^-4^ | -0.12 | 1.1x10^-5^ | -0.14 | 5.7x10^-4^ | -0.004 | NS | -0.088 | 2.7x10^-9^ | - | - | - | - | GEFOS [17] |
| 11p12 | *ARHGAP1/LRP4* | | rs7932354 | C/T | -0.24 | 9.3x10^-4^ | -0.053 | 0.029 | -0.081 | 0.025 | -0.056 | 2.4x10^-5^ | -0.073 | 1.5x10^-8^ | - | - | - | - | GEFOS [17] |
| 11q13 | *LRP5* | | rs3736228 | C/T | -0.092 | 0.33 | 0.04 | 0.27 | 0.063 | 0.19 | 0.13 | 6.3x10^-12^ | - | - | - | - | - | - | TWINS UK / ROTTERDAM [21] |
|  |  | | rs12417014 | C/T | 0.347 | 0.0088 | 0.085 | 0.019 | 0.04 | 0.45 | - | - | - | - | - | - | - | - | AOGC |
| 12q13 | *SP7* | | rs10876432 | A/G | -0.14 | 0.045 | -0.043 | 0.083 | -0.024 | 0.52 | -0.08 | 1.3x10^-7^ | - | - | - | - | -- | -- | DECODE-1 [22] |
|  |  | | rs2016266 | A/G | -0.15 | 0.026 | -0.051 | 0.035 | -0.038 | 0.31 | - | - | - | - | - | - | -0.085 | 3.1x10^-5^ | ALSPAC [34] |
| 13q14 | *RANKL* | | rs9594738 | C/T | 0.25 | 2.3x10^-4^ | 0.089 | 3.7x10^-5^ | 0.15 | 4.0x10^-6^ | -0.17 | 2.0x10^-21^ | -0.10 | 1.9x10^-8^ | - | - | - | - | DECODE-1 [23] |
|  |  | | rs9594759 | C/T | 0.09 | 0.17 | 0.042 | 0.061 | 0.11 | 0.0014 | -0.13 | 1.1x10^-16^ | -0.07 | 2.1x10^-6^ | - | - | - | - | DECODE-1 [23] |
| 14q32 | *MARK3* | | rs2010281 | A/G | -0.069 | 0.04 | -0.037 | 0.11 | 0.0 | 0.81 | - | - | -0.08 | 1.8x10^-9^ | - | - | - | - | DECODE-1 [22] |
| 16q23 | *ADAMTS18* | | rs16945612 | C/T | -0.14 | 0.21 | -0.057 | 0.15 | -0.038 | 0.31 | - | 0.016 | -0.044^a^ | 5.8x10^-7 b^ | - | - | - | - | XIONG ET AL [33]. |
|  |  | | rs11859065 | A/G | -0.13 | 0.26 | -0.054 | 0.16 | 0.063 | 0.29 | - | 0.017 | -0.044 ^a^ | 1.3x10^-6 b^ | - | - | - | - | XIONG ET AL [33]. |
|  |  | | rs11864477 | C/T | -0.13 | 0.26 | -0.054 | 0.16 | 0.063 | 0.29 | - | 0.01 | -0.044 ^a^ | 2.0x10^-6 b^ | - | - | - | - | XIONG ET AL [33]. |
| 16q24 | *FOXL1* | | rs10048146 | A/G | 0.20 | 0.020 | 0.048 | 0.085 | 0.047 | 0.25 | 0.068 | 6.0x10^-8^ | 0.012 | 8.3x10^-6^ | - | - | - | - | GEFOS [17] |
| 17q21 | *HDAC5* | | rs228769 | C/G | -0.16 | 0.045 | -0.068 | 0.013 | -0.068 | 0.090 | -0.067 | 1.0x10^-5^ | -0.081 | 5.8x10^-8^ | - | - | - | - | GEFOS [17] |
| 17q12 | *CRHR1* | | rs9303521 | G/T | 0.12 | 0.088 | 0.01 | 0.65 | 0.046 | 0.15 | 0.068 | 5.0x10^-8^ | 0.055 | 8.3x10^-6^ | - | - | - | - | GEFOS [17] |
| 17q21 | *SOST* | | rs1513670 | C/T | 0.069 | 0.30 | 0.041 | 0.083 | -0.03 | 0.34 |  |  |  |  |  |  |  |  |  |
|  |  | | rs7220711 | A/G | -0.15 | 0.019 | -0.073 | 0.0020 | 0.0 | 0.95 | - | - | -0.08 | 2.1x10^-8^ | - | - | - | - | DECODE-1 [22] |
|  |  | | rs1107748 | C/T | 0.17 | 0.011 | 0.064 | 0.0069 | 0.0 | 0.97 | - | - | -0.08 | 2.2x10^-8^ | - | - | - | - | DECODE-1 [22] |
| 18q21 | *RANK* | | rs3018362 | A/G | -0.21 | 0.0021 | -0.050 | 0.033 | -0.062 | 0.072 | - | - | -0.07 | 9.9x10^-7^ | - | - | - | - | DECODE-1 [23] |
| 20p12 | *JAG1* | | rs2273061 | A/G | -0.17 | 0.0087 | -0.043 | 0.066 | -0.047 | 0.18 | -0.072 | 5.3x10^-8^ | -0.054 | 4.2x10^-5^ | - | - | - | - | KUNG ET AL [28] |

^a^Female gwas samples. ^b^US White GWAS Sample
